# From signalling pathways to targeted therapies: unravelling glioblastoma’s secrets and harnessing two decades of progress

**DOI:** 10.1038/s41392-023-01637-8

**Published:** 2023-10-20

**Authors:** Brittany Dewdney, Misty R. Jenkins, Sarah A. Best, Saskia Freytag, Krishneel Prasad, Jeff Holst, Raelene Endersby, Terrance G. Johns

**Affiliations:** 1 Cancer Centre, Telethon Kids Institute, Nedlands, WA 6009 Australia; 2 Centre For Child Health Research, University of Western Australia, Perth, WA 6009 Australia; 3 Immunology Division, The Walter and Eliza Hall Institute of Medical Research, Melbourne, 3052 Australia; 4 Department of Medical Biology, University of Melbourne, Melbourne, 3010 Australia; 5 Personalised Oncology Division, The Walter and Eliza Hall Institute of Medical Research, Melbourne, 3052 Australia; 6 School of Biomedical Sciences, University of New South Wales, Sydney, 2052 Australia

**Keywords:** CNS cancer, Tumour heterogeneity, Drug development

## Abstract

Glioblastoma, a rare, and highly lethal form of brain cancer, poses significant challenges in terms of therapeutic resistance, and poor survival rates for both adult and paediatric patients alike. Despite advancements in brain cancer research driven by a technological revolution, translating our understanding of glioblastoma pathogenesis into improved clinical outcomes remains a critical unmet need. This review emphasises the intricate role of receptor tyrosine kinase signalling pathways, epigenetic mechanisms, and metabolic functions in glioblastoma tumourigenesis and therapeutic resistance. We also discuss the extensive efforts over the past two decades that have explored targeted therapies against these pathways. Emerging therapeutic approaches, such as antibody-toxin conjugates or CAR T cell therapies, offer potential by specifically targeting proteins on the glioblastoma cell surface. Combination strategies incorporating protein-targeted therapy and immune-based therapies demonstrate great promise for future clinical research. Moreover, gaining insights into the role of cell-of-origin in glioblastoma treatment response holds the potential to advance precision medicine approaches. Addressing these challenges is crucial to improving outcomes for glioblastoma patients and moving towards more effective precision therapies.

## Introduction

Glioblastoma is a WHO grade IV adult-type, isocitrate dehydrogenase (IDH)-wildtype, diffuse astrocytic glioma.^1^ Although glioblastoma is a relatively rare diagnosis with 3–5 cases per 100,000 people, it is the most common form of high-grade glioma (HGG). Furthermore, the prognosis of glioblastoma remains one of the worst in clinical oncology, with an average overall survival (OS) of 12–15 months.^2,3^ Less than 5% of glioblastoma patients survive 5 years after their initial diagnosis and this rate has hardly improved over the past century.^4–7^ Most diagnoses occur in older adults from Westernised countries, with males having a higher incidence rate.^8,9^

Primary glioblastoma develops spontaneously, whereas secondary glioblastoma, which accounts for roughly 10% of cases, occurs more frequently in younger patients and may develop from the progression of lower grade astrocytomas. There is little evidence of hereditary or environmental causes specific for glioblastoma. Radiation exposure is the only confirmed environmental risk factor for all brain tumours, including glioblastoma.^10–12^ A number of genetic predispositions are specifically associated with increased risk of glioblastoma versus non-glioblastoma glioma, including single nucleotide polymorphisms (SNPs) in ribonucleoprotein, PTB binding 2 (*RAVER2*), epidermal growth factor receptor (*EGFR*), HEAT repeat-containing protein 3 (*HEATR3*), solute carrier family 16 member 8 (*SLC16A8*), telomerase reverse transcriptase *(TERT)*, cyclin-dependent kinase inhibitor 2B (*CDKN2A/B*) and regulator of telomere elongation helicase 1 (*RTEL1*).^13^ As a highly aggressive, heterogeneous, and undifferentiated brain tumour, the pathogenesis of primary glioblastoma is poorly understood. The critical location and rarity of these tumours makes it difficult to fully understand the mechanisms behind their development. Until recently glioblastoma was considered a lump of tissue in the brain. We now know that the tumour microenvironment is unique in glioblastoma as the cells are surrounded by electrically active neurons, microglia, and astrocytes that support glioblastoma growth.^14,15^ These distinctive characteristics, as well as the presence of the blood–brain barrier (BBB), make glioblastoma challenging to treat. These factors need to be considered to advance our understanding of tumour biology and response to treatment. This, among other challenges, are currently major barriers that have prevented the success of targeted therapies explored in the clinic.^16^

As our understanding of the mechanisms behind glioblastoma pathogenesis continues to emerge, various targeted approaches have been explored over the last few decades. While the vast majority of glioblastoma clinical research has focused on therapeutic targeting of receptor tyrosine kinase (RTK) signalling pathways, other avenues for targeted therapy include epigenetics, metabolism and immune-targeted therapies. This article will review what is currently known about glioblastoma epidemiology, pathogenesis, and treatment, after more than 100 years of research. We will also discuss the most promising avenues for targeted therapies to treat glioblastoma. The scope of this review will focus mostly on the perspective of glioblastoma, but we will occasionally include relevant discussion on other HGGs (grade III or IV) more broadly, particularly paediatric grade IV gliomas. The use of molecular biomarkers in diagnosing central nervous tumours (CNS) has led to significant changes in brain tumour classifications in recent years, particularly around grouping of diffuse gliomas based on IDH mutation status.^1,17^ For example, glioblastoma is now exclusively used for diagnosis of adult IDH-wildtype tumours, and no longer includes IDH-mutant tumours or paediatric tumours.^1^ For the purposes of this review, the term glioblastoma is still used to describe grade IV IDH-wildtype or IDH-mutant glioblastomas as they were defined in the relevant cited works, which may not have used the updated WHO classification system (5th edition 2021). Furthermore, grade IV paediatric HGGs are now classified as one of the following based on IDH status and histone 3 (H3) status: diffuse paediatric-type HGG IDH-wildtype and H3-wildtype, diffuse midline glioma (DMG) H3K27-altered, diffuse hemispheric glioma H3G34-mutant, or infant-type hemispheric glioma.^1^ Although the term paediatric glioblastoma is no longer applicable based on this classification system, we will use the term paediatric glioblastoma in this review where the original research work cited used the term to describe non-DMG grade IV paediatric gliomas. The term DMG will be used to describe paediatric H3K27-mutant gliomas, as well as cases that were previously referred to as diffuse intrinsic pontine glioma.

### Historical milestones and perspectives on glioblastoma classification

Glioma was first described in 1865 by Rudolf Virchow who identified malignant tumours that he believed to originate from glial cells on post-mortem histological examination.^18^ Heinrich Sroebe later confirmed the infiltrative nature of gliomas and introduced the concept of histological subtypes to explain the intratumoural heterogeneity observed.^18^ To this day, this remains as one of the most significant discoveries for understanding glioblastoma pathogenesis and highlights the importance of multifocal examination of brain tumours. Of course, with considerable advances in technology, we can now characterise intra- and intertumoural heterogeneity on the molecular level, rather than only by macroscopic and histological examination.

The first confirmed craniotomy for the resection of a brain tumour was performed by Rickman Godlee in 1884. The field of neurosurgery then blossomed in the 1900s, beginning with Harvey Cushing’s revolutionary surgical technique that incorporated subtemporal decompression to control intracranial pressure. By 1910, Cushing had established this technique as the first surgical advancement to substantially reduce operative mortality.^19,20^

Classification of brain tumours began after Globus and Strauss proposed the name ‘spongioblastoma multiforme’ in 1925 for the unique clinical and histological characteristics of a subtype of malignant glioma.^21^ Bailey and Cushing challenged this terminology in 1926 and suggested a classification system for brain tumours based on their cell-of-origin, leading to the suggestion of the originally used term, ‘glioblastoma multiforme’.^22^

Alongside the argument over which terminology should be used, there was also continued debate throughout the early 1900s over the practice of surgical resection for malignant brain tumours due to the associated risks and complications in finding the lesions and the lack of improvement in clinical outcomes. The introduction of ventriculography, the Potter-Bucky X-ray Grid, and angiography improved localisation of tumours and visualisation of vasculature, rapidly advancing surgical practices.^23,24^ However, it wasn’t until the late 1950s that two milestone clinical studies demonstrated the relevance of the degree of surgical resection on patient survival and introduced the beneficial role of adjuvant radiotherapy (RT).^25,26^ Despite ongoing efforts to improve therapeutic options, the next landmark in glioblastoma treatment did not occur until 1997 with the discovery of the oral alkylating agent temozolomide (TMZ).^27^ Over the succeeding decade, work by Roger Stupp and colleagues showed that concomitant TMZ and RT significantly improved OS and progression-free survival (PFS) compared with RT alone, albeit only by a couple of months.^2,28^

More recently, technological advances, particularly the continued evolution of genetic sequencing capabilities, has facilitated a deeper understanding of glioblastoma pathobiology. The inundation of ‘big data’ in recent years has improved our knowledge regarding the cell-of-origin of glioblastoma tumours, and therefore, has changed the way we think about the classification of glioblastoma. Over the past 15 years, tumour cell states in glioblastoma have been intensively investigated. While initial bulk sequencing studies identified the most highly prevalent glioblastoma mutations,^29^ research focus has since evolved into developing a framework for glioblastoma subtypes.

Three subtypes—proneural, mesenchymal, and classical—have been identified based on gene expression and copy number changes.^30–32^ Key alterations specific to each subtype, such as neurofibromatosis type 1 (*NF1)* in the mesenchymal subtype, epidermal growth factor receptor (*EGFR)* in the classical subtype and platelet-derived growth factor receptor α (*PDGFRA)* in the proneural subtype, have provided clues to their cell-of-origin. For example, the proneural subtype is enriched for the oligodendrocyte development gene signature. Therefore, this subtype may arise from a progenitor or neural stem cell in the oligodendrocyte lineage, such as PDGFRA-positive cells in the sub-ventricular zone, one of the main neurogenic regions in the adult brain.^31,33^ Bulk transcriptomics have enabled the prediction of intra-tumoural heterogeneity, allowing researchers to deconvolute an individual tumour to estimate the mixture of glioblastoma subtypes. This method also highlights any differences in cellular composition between the tumour core and the tumour periphery.^34^

More recent studies have moved away from traditional bulk transcriptomic analysis to single cell RNA sequencing (scRNAseq) analyses,^35,36^ which allow the transcriptional characterisation of thousands of individual cells from a single biological sample. This technology has been instrumental in redefining subtypes in the context of the normal brain. Recently, an scRNAseq study compared the human foetal brain with glioblastoma samples to define tumour subtypes using direct parallels with normal developmental cell states.^36^ These tumour cell states include neural progenitor cell-like (NPC-like), astrocyte-like (AC-like), oligodendrocytic precursor cell-like (OPC-like), each of which closely parallel normal cell states, and mesenchymal-like (MES-like), which has similarities to radial glia, but is more distantly associated with normal glial cells.^35,36^ These studies provide the strongest evidence to date defining the glial cell origins of glioblastoma in patient samples. Furthermore, scRNAseq has also shown that tumour composition is not only variable across patients but is immensely heterogeneous within a single tumour.^32,35,36^ Recent longitudinal studies following treatment exposure have also shown that the proportion of tumour cells within each subtype is plastic and changes as a result of therapeutic insult.^37^ Specifically, the prevalence of the mesenchymal subtype may increase due to a higher portion of cycling mesenchymal cells following therapy.^37^ The implications of this knowledge for future targeted therapies in glioblastoma will be discussed later in this review.

Unfortunately, the considerable technological advances and improved knowledge of glioblastoma subtypes gained over the past century have not translated into improved patient outcomes. The average survival of glioblastoma patients in the 19^th^ century was very similar to the survival outcomes we see in patients today.^25,26^ Therefore, we urgently need more effective targeted therapies to improve the outcomes and quality of life of glioblastoma patients.

## Current treatment options

Standard glioblastoma treatment has not changed since 2005, and involves surgical resection, followed by radiation and concurrent and adjuvant TMZ.^2^ The addition of adjuvant TMZ to clinical protocols resulted in a marginal increase in survival time for adults with glioblastoma.^2,6^ However, patient quality of life is decreased due to the systemic toxicities caused by this chemotherapeutic agent. Moreover, many tumours show primary resistance to temozolomide or acquire resistance during the treatment regime.^38^ The approval of the anti-angiogenic antibody, bevacizumab, which neutralises vascular endothelial growth factor (VEGF), for the treatment of recurrent glioblastoma lead to initial excitement as it resulted in an increase in PFS in patients.^39^ However, while subsequent trials also reported an increase in PFS, there was no increase in OS for newly diagnosed patients.^40^ Available data suggest that bevacizumab normalises the blood vessels within the tumour, leading to a reduction in symptoms such as oedema, but does not extend lifespan.^41^ Due to the widely infiltrative nature of glioblastoma and its intrinsic resistance to standard therapies, glioblastoma remains non-curative to this day and urgently requires new therapeutic options.

## RTKs in glioblastoma

**Fig. 1:**
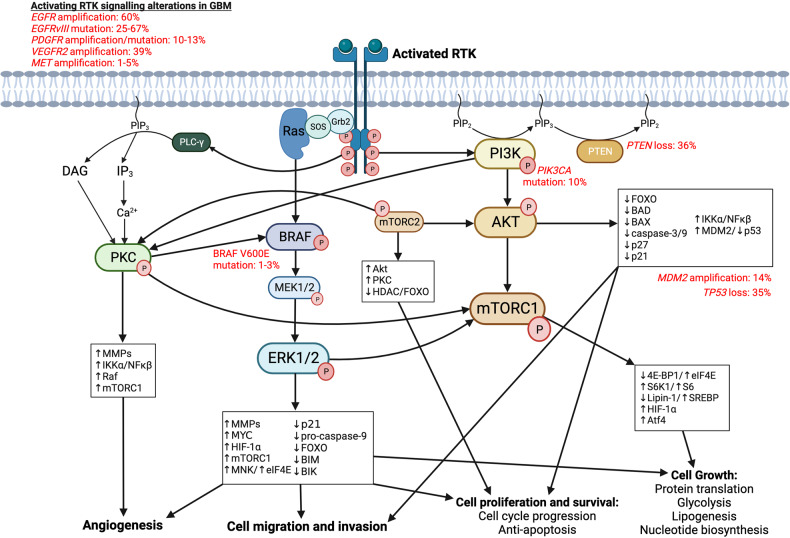
RTK signalling pathways in glioblastoma. RTKs have been identified as activating oncogenes that promote glioblastoma tumourigenesis, including epidermal growth factor receptor (EGFR), platelet derived growth factor receptor (PDGFR), vascular endothelial growth factor receptor (VEGFR), fibroblast growth factor receptor (FGFR), and hepatocyte growth factor receptor (HGFR or c-Met). The main signalling cascades activated by any of the above RTKs include PI3K/AKT/mTOR and Ras/Raf/MEK/ERK. Activation of these cascades regulates proteins and transcription factors, such as HIF-1α, FOXO, and NFκβ, that promote cell and cycle progression, cell growth and survival, cell migration and invasion, and angiogenesis. Importantly, mTORC2 and PKC can act as overlapping relay signalling kinases. Independently mTORC2 and PKC can phosphorylate and regulate proteins required for tumourigenesis, however, they also play an important role in stimulating positive feedback loops within the PI3K/Akt/mTORC1 and Ras/Raf/MEK/ERK cascades. mTORC2 activates AKT, which can subsequently increase mTORC1 activity for promoting cell growth and metabolism. In addition, mTORC2 also activates PKC; downstream targets of PKC include mTORC1 and Raf, therefore, mTORC2 and PKC may dependently or independently contribute to sustained kinase signalling in glioblastoma cells. This crosstalk between pathways allows glioblastoma cells to easily overcome any blocks by targeted therapies, complicating our understanding of the clinical implications in glioblastoma treatment management and therapeutic resistance. Figure created with Biorender.com

A number of complex and interconnected cell signalling cascades have critical roles in cancer hallmarks, including sustained proliferative signalling, resisting cell death, replicative immortality, and invasion and metastasis. Most discovery and development programmes for targeted glioblastoma therapies have focused on small molecule inhibitors and antibodies directed at RTKs and relevant downstream signalling pathways. All RTKs have the same generic structure, including an extracellular ligand-binding domain, a single transmembrane helix that spans the plasma membrane and an intracellular domain containing the protein tyrosine kinase domain. In general, upon activation by ligand binding, RTKs stabilise into their active dimeric/oligomeric form, which disrupts the ‘cis’-autoinhibition within the active loop of the kinase domain and leads to activation of tyrosine kinase activity.^42^ The kinase activity then drives intracellular signalling cascades that are key regulators of normal cell processes, such as growth. Therefore, alterations in RTKs directly contribute to the oncogenic intracellular signalling pathways that drive cancer cell progression. Several RTKs have aberrant expression, mutations, or copy number alterations in glioblastoma. The most frequently affected RTKs are epidermal growth factor receptor (EGFR), platelet-derived growth factor receptor (PDGFR), vascular endothelial growth factor receptor (VEGFR), fibroblast growth factor receptor (FGFR), and hepatocyte growth factor receptor (HGFR or c-Met) (Fig. 1).

### EGFR

EGFR is a single-pass transmembrane receptor tyrosine kinase.^43^ The extracellular region of EGFR contains four separate domains (I, II, III and IV). Ligands, such as EGF and TGFα, interact with domains I and III, resulting in non-covalent receptor dimerisation and activation.^43^ EGFR is mutated, rearranged, alternatively spliced and/or focally amplified in approximately 60% of glioblastoma tumours.^32^ Since these alterations appear to be oncogenic drivers in glioblastoma, EGFR is a prime target for glioblastoma therapy.^32,44,45^ The constitutively active deletion mutant, EGFR variant III (EGFRvIII), is among the most common alterations found in glioblastoma, occurring in around 30% of glioblastoma patients.^46^ The deletion removes most of domain I and a large part of domain II, rendering EGFRvIII unable to bind ligands.^46^ The deletion also generates a free cysteine that enables covalent EGFRvIII dimerisation and autophosphorylation.^47^ EGFRvIII is a bona fide cancer-specific target as it is not expressed in normal tissue.^46^ Interestingly, EGFRvIII is activated at a low level, allowing it to evade internalisation and downregulation, thus leading to sustained signalling.^48^

*EGFR* amplification leads to overexpression of the EGFR at the cell surface, which in turn leads to autoactivation of the EGFR.^44^ A range of different missense mutations, mostly in the extracellular domain,^32,44^ are also found in EGFR in glioblastoma, which is different to non-small cell lung cancers where EGFR missense mutations are more commonly found in the tyrosine kinase domain. In glioblastoma, these mutations are nearly always found in tandem with amplified *EGFR* and are autoactivating via varied mechanisms.^32,44,47,49^

### PDGFR

The PDGFR family includes PDGFRα and PDGFRβ, which can both exist as homodimers or heterodimers, c-KIT (also known as stem cell factor receptor/CD117) and Fms-like tyrosine kinase 3 (FLT3).^42^ The ligand for PDGFRα/β, PDGF, arises from four gene products, *PDGFA*, *PDGFB*, *PDGFC* and *PDGFD*, and forms either homodimers (PDGF-AA/BB/CC/DD) or a heterodimer (PDGF-AB).^50^ Binding of PDGF-AA/BB/AB/CC encourages PDGFRα homodimerisation, PDGF-BB/DD promotes PDGFRβ homodimerization, and PDGF-AB/BB/CC/DD encourages PDGFRαβ heterodimersation.^51^ PDGFRα is essential for embryonic development, particularly oligodendrocyte development,^52^ while PDGFRβ plays an essential role in blood vessel formation.^53,54^

The gene for PDGFRα, *PDGFRA*, is significantly amplified in 10–13% of glioblastoma cases.^29,31,32^ In addition, *PDGFRA* gene rearrangements have been identified in *PDGFRA*-amplified glioblastoma. For instance, the *PDGFRA*^*Δ8,9*^ mutation, characterised by an in-frame deletion within exons 8 and 9, gives rise to a transformed oncogenic receptor with ligand-independent receptor activation.^55^ Considered to be the PDGFR equivalent of EGFRvIII, this mutant form is often co-expressed with wildtype *PDGFRA* in *PDGFRA*-amplified tumours.^55^ *PDGFRB* mutation/amplification is less commonly found, but may be preferentially expressed in glioma stem cells or in the proliferating glioblastoma microvasculature.^56,57^ In contrast, *FLT3* is often found to have copy number loss in glioblastoma.^58^ c-Kit expression is variable and mutations are rare in glioblastoma, however, some studies have reported high-level amplification of *KIT* in roughly 30% of glioblastoma tumours.^59,60^

### FGFR

Four different FGFRs (FGFR1, FGFR 2, FGFR3 and FGFR4) have been characterised and are well known for their importance in neuronal development and neural stem-cell maintenance.^61,62^ They are activated by the FGF family, which is the largest family of growth factor ligands, comprising 22 ligands in humans.^63^ *FGFR* amplification is found in only 3.2% of glioblastoma cases,^32^ and is therefore, less common than *PDGFRA* amplification. However, in some glioblastoma patients, chromosomal translocations give rise to *FGFR* gene fusions between *FGFR1* or *FGFR3* and transforming acidic coiled-coil (TACC) proteins, generating FGFR-TACC fusion proteins that exhibit constitutively active kinase activity in glioblastoma cells.^64^ Furthermore, FGFR3 is significantly upregulated in infiltrating glioblastoma cells within the tumour periphery.^65^ In *IDH*-wildtype/*FGFR*-mutant glioblastomas, *FGFR3* is the most commonly altered *FGFR* gene, including amplification and *FGFR3-TACC3* fusions.^66^

In glioblastoma cases from The Cancer Genome Atlas (TCGA), *FGFR2* is most commonly deleted and several *FGFR2* fusion genes have been identified, including *CXCL17–FGFR2*, *SIPA1L3–FGFR2*, *FGFR2–SIPA1L3* and *FGFR2–CEACAM1*.^67^ However, these alterations did not produce functional proteins due to a disruption in the FGFR2 kinase domain and are thus unlikely to contribute to oncogenic activity.^67^ Intriguingly, a recent case report has identified the first case of *FGFR2* amplification with a novel *FGFR2-TACC2* fusion protein in a glioblastoma patient with an aggressive *IDH*-mutant tumour.^68^ Since *FGFR* alterations have not been previously shown to occur in *IDH*-mutant glioblastomas, this curious phenotype warrants further investigation for its role in glioblastoma and potential targetability.

### HGFR/c-Met

c-Met is a single RTK activated by the HGF ligand that is normally responsible for skeletal muscle growth during embryonic development.^69^ High levels of HGF are secreted by glioblastoma cells, likely through EGFRvIII signalling.^70,71^ Thus, EGFRvIII-mediated secretion of HGF creates an important autocrine signalling loop where EGFRvIII transactivates c-Met and plays a critical role in glioblastoma stem cell maintenance.^72,73^ Furthermore, c-Met expression may be induced by EGFR inhibition and is associated with therapeutic resistance as it promotes a population with a high stemness phenotype.^74,75^ c-Met is believed to elicit similar downstream signalling cascades to EGFR and co-localises with EGFR expression in glioblastoma tumours.^76^ Thus, the cross-talk between c-Met and EGFR/EGFRvIII likely plays a significant role in driving an aggressive and malignant glioblastoma phenotype. However, *MET* amplification only occurs in 1–5% of glioblastomas.^29,32,77^

### VEGFR

*VEGFR* mutations and copy number variations are not commonly found in genetic signatures of glioblastoma. Although *VEGFR2* gene copy numbers have been reported to be amplified in 39% of glioblastomas,^60^ this was from a single small population study that has not been validated in larger glioblastoma cohorts. However, VEGFR expression plays a significant role in tumour pathogenesis and is often increased in glioblastoma tumours in response to hypoxia, contributing to increased angiogenesis and irregular tumour vasculature. Three VEGF receptors have well-established roles in regulating vasculogenesis during embryonic development, as well as angiogenesis in both normal and neoplastic tissue: VEGFR-1 (activated by VEGF-A, VEGF-B and placental growth factor); VEGFR-2 (activated by VEGF-A, VEGF-C, VEGF-D, VEGF-E and VEGF-F) and VEGFR-3 (activated by VEGF-C and VEGF-D). There is strong evidence supporting the role of VEGFR2 expression in the development of glioblastoma, including the promotion of tumour cell survival, invasion, and therapeutic resistance through autocrine signalling.^78–83^ While VEGFR2 expression may vary in all types of primary glioma,^84^ it appears that VEGFR expression may have an underestimated role within glioblastoma genetic signatures.

## RTK downstream signalling pathways

### Phosphatidylinositol 3-kinase (PI3K)/v-akt murine thymoma viral oncogene homologue (AKT)/mammalian target of rapamycin (mTOR)

RTK activation by autophosphorylation of tyrosine residues stimulates the PI3K/AKT/mTOR signalling cascade, an important pathway responsible for regulating cell survival, cell proliferation, and metabolism, especially in high-stress environments, such as tumourigenesis. The PI3K axis is especially significant to glioblastoma pathogenesis, since as many as 86% of glioblastomas harbour a genetic alteration in the core RTK/PI3K pathway.^29^ RTK activation recruits PI3K to the plasma membrane. PI3K is a lipid protein kinase that phosphorylates phosphatidylinositol. Most relevant to glioblastoma cell signalling is class I PI3Ks, which form heterodimers consisting of a catalytic p110 subunit (p110α, p110β, p110δ or p110γ) and a regulatory subunit (p85α, p85β, p55γ, p101 and p84). The p110α, p110β and p110δ subunits, which make up class IA PI3Ks, are differentially expressed in glioblastoma.^85^ Mutations in *PIK3CA*, the gene that encodes p110α, have been described in about 10% of glioblastoma cases.^86^ Notably, p110β expression has been associated with poor survival in glioblastoma patients and has been shown to promote glioblastoma cell survival and AKT activation in vitro.^85^ PI3K activation leads to p110-mediated conversion of phosphatidylinositol-4,5-bisphosphate (PIP_2_) to phosphatidylinositol-3,4,5-triphosphate (PIP_3_). PIP_3_ recruits additional signalling protein kinases to the membrane via their pleckstrin homology (PH) domains, including protein serine/threonine kinase-3′-phosphoinositide-dependent kinase 1 (PDK1) and AKT, a known master regulator of intracellular signalling in tumourigenesis. There are three AKT isoforms, AKT1, AKT2 and AKT3, which may play functionally distinct functions in cell survival, glucose metabolism, and neuronal development, respectively.^87^

Evasion of cell death and sustained proliferative signalling are two of the original hallmarks of cancer described by Hanahan and Weinberg.^88^ AKT activation regulates cell cycle progression and apoptosis through several mechanisms, including by phosphorylating and inactivating the forkhead box class O (FOXO) transcription factor family, thus reducing expression of pro-apoptotic and cell cycle-inhibitory genes.^89,90^ When activated, FOXO transcription factors act as tumour suppressors, which tightly regulate cellular homoeostasis through regulation of apoptosis, cell cycle arrest, metabolic pathways and response to oxidative stress.^90,91^ PI3K signalling and AKT-mediated inactivation is undoubtedly one of the most important regulatory mechanisms controlling FOXO nuclear localisation and transcriptional activity. In addition, AKT activation leads to apoptosis evasion through phosphorylation and inhibition of pro-apoptotic proteins BAD, BAX, and caspase-3/9.^89,92^

Another downstream target of AKT is MDM2 (mouse double minute 2 homologue), an oncoprotein that, upon phosphorylation by AKT, translocates to the nucleus to bind p53 and target the tumour suppressor for degradation;^93^ yet another mechanism by which AKT inhibits cellular apoptosis. Loss of p53 signalling, the most common oncogenic phenotype documented in all human cancers, is altered in up to 87% of glioblastoma patients. *MDM2* amplification occurs in 14% and *TP53* is mutated or deleted in 35% of glioblastomas.^29^ Moreover, AKT activation promotes cell cycle progression through phosphorylation and inhibition of cell cycle inhibitors p27 and p21, leading to stabilised cyclin D1/D3 and cell growth. AKT also phosphorylates and inhibits glycogen synthase kinase-3β,^94^ which is another modulator of multiple signalling and metabolic proteins, and may play both oncogenic and tumour suppressor roles in cancer.^95^

Finally, AKT phosphorylates IKKα (ΙκB kinase), subsequently leading to activation of the oncoprotein nuclear factor kappa B (NF-κβ). NF-κβ activity also plays a broad oncogenic role, including regulating transcription of anti-apoptosis genes, promoting expression of cyclins for cell cycle progression, and inducing expression of cell invasion and angiogenesis proteins, such as matrix metalloproteinases (MMPs) and VEGF.^96^ Furthermore, NF-κβ suppresses expression of the well-known tumour suppressor *PTEN*,^97^ a negative regulator of PI3K signalling that converts PIP_3_ back to PIP_2_. PTEN loss (either by deletion or mutation), which leads to constitutive PI3K/AKT pathway activation, is found in 36% of glioblastomas^29^ and is associated with aggressive tumour growth and poor patient survival.^98^

While AKT activation regulates several cellular processes on its own as a key mediator of cancer cell transformation, AKT is also responsible for activating another master regulator, mTORC1. mTORC1 regulates cell growth by promoting protein translation,^99^ lipogenesis,^100^ and nucleotide biosynthesis.^101^ mTORC1 phosphorylates and activates ribosomal protein S6 kinase (S6K1) and subsequently S6, a ribosomal protein that is part of the 40S subunit required for protein translation.^102^ In addition, mTORC1 phosphorylates and inhibits eukaryotic initiation factor 4E (eIF4E)-binding protein (4E-BP1), allowing for exposure of 5’ cap-associated factor eIF4E, followed by recruitment of the 40S ribosomal machinery required for translation initiation.^99^ In a nutrient- and growth factor-rich environment, mTORC1 promotes cell growth by phosphorylating and inhibiting lipin-1. This prevents downregulation of sterol regulator element binding protein (SREBP) transcription factors and increases expression of fatty acid synthase, promoting lipid synthesis, which is essential for generating membranes during cancer cell growth and proliferation.^100^

Similarly, mTORC1 also activates hypoxia-inducible factor 1α (HIF1α),^103^ particularly in *PTEN*-deficient tumours,^104,105^ although the mechanisms by which this occurs remain unclear. HIF1α has well-established roles in regulating processes such as angiogenesis, another classical cancer hallmark, as well as in inducing glycolysis,^106,107^ characteristic of the ‘Warburg effect’ related to metabolic reprogramming, an emerging cancer hallmark introduced in 2011.^88^ Finally, mTORC1 increases metabolic flux of one-carbon units from the mitochondrial tetrahydrofolate cycle into the de novo purine synthesis pathway through increased expression of activating transcription factor 4 and methylenetetrahydrofolate dehydrogenase 2.^101^ Although this is only a brief summary of the mechanisms in which mTORC1 regulates cancer cell growth, these roles are well-established in tumour development.

Upstream regulation of the second mTOR complex, mTORC2, is less well understood, although it may be activated by PI3K signalling and ribosome binding.^108^ mTORC2 is most commonly known for its ability to phosphorylate and activate AKT,^109^ resulting in overlapping positive feedback loops within the PI3K pathway that can be a major oncogenic driver and mechanism of resistance to PI3K/AKT/mTOR inhibition in glioblastoma. mTORC2 has emerging roles in metabolic reprogramming in glioblastoma by modulating glycolysis, lipid metabolism, and glutamine metabolism, mediated by AKT and MYC. Independently of PI3K/AKT signalling, EGFRvIII-mediated activation of mTORC2 phosphorylates and inhibits class IIa histone deacetylase complexes (HDACs), leading to deacetylation of FOXO transcription factors.^110^ Inhibition of FOXO activity results in upregulation of MYC, reduced gluconeogenesis, and increased glycolysis.^110^ Thus, the RTK/ PI3K/AKT/mTOR pathway elegantly influences a range of processes including cell cycle regulation, metabolism, and cell growth (Fig. 1), as well as linking other regulatory epigenetic mechanisms (see Targeting epigenetic pathways below).

### Mitogen-activated protein kinase (MAPK) cascade: Ras/Raf/MEK/ERK

MAPK cascades are well-established as extracellular signal transduction pathways that regulate cellular processes. The ERK signalling pathway is the most well-established MAPK cascade relevant to RTK signalling and tumourigenesis, and has been well described in countless previous reviews.^111–113^ In brief, activation of RTKs leads to recruitment of adaptor proteins and guanine nucleotide exchange factors, such as growth factor receptor bound protein 2 (Grb2) and son of sevenless protein (SOS), followed by recruitment of the GTP-coupled protein, Ras. Activation of Ras at the plasma membrane begins the MAPK cascade, starting with the first MAPKKK effector Raf. Of the Raf protein family, BRAF is most frequently implicated in cancer development. The most common *BRAF* mutation is a class I point mutation, *BRAF* V600E. This is the only *BRAF* mutation found in glioblastoma, albeit at a very low prevalence. Only 1–3% of patients harbour the *BRAF* V600E mutation, but it is more frequent in children and young adults and in epithelial-type glioblastoma.^114–117^ Mutated *BRAF* causes constitutive activation of BRAF, leading to continued activation of its downstream effector MAPKs, MEK1/2 and ERK1/2. Activated MEK/ERKs act as master regulators of cell proliferation, survival, and growth by regulating transcription factors required for cell cycle progression,^111,118^ negatively regulating expression of pro-apoptotic genes and proteins,^118,119^ activating RNA polymerase I/III transcription factors, and therefore, expression of ribosomal protein genes^120–122^ and regulating glycolytic flux, likely through activation of the transcription factor MYC.^123,124^ The latter two outcomes strongly overlap with PI3K/AKT/mTOR signalling, as ERKs can directly phosphorylate and activate mTORC1.^125,126^ Moreover, ERK and AKT/mTOR share similar downstream targets, including MYC and HIF1α, and therefore, work in synergy to evoke a response that supplies cancer cells with the proteins and energy required for growth and active proliferation (Fig. 1).

More specifically, both p38 and ERK1/2 MAPKs are known to activate MAPK-interacting kinase (MNK) and subsequent phosphorylation of eIF4E.^127^ As discussed above, the phosphorylation of 4E-BP1 by mTORC1 removes the bind from eIF4E, which allows for phosphorylation of eIF4E by MNK to promote protein translation initiation. Thus, the divergence of the MAPK/ERK and AKT/mTOR signalling cascades plays a significant role on regulating the MNK-eIF4E axis to regulate mRNA translation.^128^ This event is critical for tumourigenesis as phosphorylated eIF4E (p-eIF4E) has been shown to be required for translation of pro-oncogenic factors in glioma cells including MMPS, VEGF, and B-cell lymphoma 2 (Bcl-2), which are responsible for cancer cell invasion, angiogenesis, and anti-apoptotic mechanisms, respectively.^129^ Furthermore, total levels of eIF4E and p-eIF4E expression are positively associated with tumour grade.^130^ Strikingly, p-eIF4E expression is highly sensitive for diagnosing glioblastoma and is an independent predictor of survival in glioma patients.^130^ The consideration of eIF4E as a therapeutic target in glioblastoma is still in its infancy and is relatively unexplored. Only very recently has a pre-clinical study shown for the first time that tomivosertib, an MNK1 inhibitor, was effective at inhibiting glioblastoma cell growth and angiogenesis via reduced p-eIF4E levels.^131^ In addition, tomivosertib prevented TMZ-induced activation of eIF4E, suggesting that the combination of tomivosertib with TMZ could enhance therapeutic efficacy and prevent treatment resistance in glioblastoma.^131^ Collectively, the MNK/eIF4E axis and combined activating roles of MAPK/ERK and AKT/mTOR signalling warrants further exploration for understanding this new potential therapeutic target in future functional studies and clinical trials. As the extensive crossover of the MAPK and PI3K/AKT/mTOR signalling cascades have been described thus far, it is no wonder that targeting broad effector molecules such as AKT, mTOR, or BRAF/MEK have proven difficult as a treatment strategy in glioblastoma. A more focused approach should be considered by including more specific targeted therapies, such as inhibiting protein translation by blocking p-eIF4E.

### Phospholipase C (PLC)-γ/protein kinase C (PKC)

Activated RTKs may also phosphorylate PLC-γ, which catalyses a reaction with PIP_2_ at the plasma membrane, producing diacylglycerol (DAG) and inositol 1, 4, 5-triphosphate (IP_3_), and consequently, increasing intracellular calcium. PKC is a crucial relay kinase, with 13 isoforms that are classified based on their activation, which can be either dependent or independent on the presence of DAG, phosphatidylserine, and intracellular calcium. Due to their variability in terms of expression, activity, and range of downstream targets, the precise role of PKCs in glioblastoma pathogenesis remains poorly understood. Nevertheless, several PKC isoforms are known to regulate multiple cellular processes in cancer, including angiogenesis, proliferation, apoptosis, and invasion, often indirectly through downstream regulation of any number of the modulators within the PI3K or MAPK cascades. As another feedback mechanism, mTORC2 may also activate PKC, promoting its kinase activity.^132,133^

PKCα plays a role in glioma cell proliferation and survival, either through EGFR/mTORC signalling,^134^ ERK1/2^135^ or activation of NF-κβ.^136^ PKCβ is activated in response to hypoxia and plays a pivotal role in regulating angiogenesis in brain endothelial cells.^137,138^ Therefore, PKCβ is likely a contributing factor to the irregular vasculature formed during glioblastoma development and has been of interest as a therapeutic target.^139^ Other members of the PKC family with demonstrated roles in glioblastoma pathogenesis include PKCδ,^140,141^ PKCε,; ^142–145^ PKCη,; ^146–148^ PKCλ/ι; ^149,150^ and PKCζ. ^151,152^ Thus, while the PI3K and MAPK cascades can individually promote glioblastoma tumour development and progression, PKC signalling overlaps with both of these pathways, demonstrating the deeply interconnected and sophisticated signalling network expertly exploited by glioblastoma cells (Fig. 1).

## Targeting RTKs in glioblastoma

### Small molecule multi-target RTK inhibitors

At least 89 small-molecule inhibitors have been approved by the FDA for use in clinical oncology, and this list continues to grow.^153,154^ Since the approval of TMZ as a first-line treatment for glioblastoma, dozens of small molecule inhibitors have been extensively tested and trialled in patients in attempt to improve clinical outcomes (See Table 1 for a comprehensive list of glioblastoma clinical trials on targeted therapies against RTKs and downstream RTK signalling pathways). Imatinib was the very first small molecule inhibitor approved by the FDA in 2001 for use in chronic myelogenous leukaemia (CML).^154,155^ Imatinib targets PDGFR, BCR-ABL and c-KIT, making it one of several multi-targeting RTK inhibitors on the market, and is the gold-standard for treating CML and gastrointestinal stromal tumours (GISTs). In the context of glioblastoma, imatinib has been trialled mostly in patients with recurrent disease either as a single agent,^156,157^ in combination with hydroxyurea,^158–161^ or in combination with traditional TMZ^162^ or RT.^163^ Although the toxicity of imatinib in glioblastoma patients was tolerable in clinical studies, the overall clinical benefit of imatinib is limited in glioblastoma patients. Imatinib shows no efficacy as a single agent therapy. A small phase II study of imatinib in combination with hydroxyurea, which blocks DNA synthesis and cell cycle progression by inhibiting ribonucleotide reductase,^164,165^ showed promising results. This study demonstrated a median OS of 48.9 weeks in recurrent glioblastoma patients treated with imatinib and hydroxyurea.^158^ However, these outcomes were not observed in a larger-scale multi-centre phase II study where the median PFS was only 5.6 months,^160^ which is comparable to standard RT and TMZ treatment. Furthermore, comparing newly diagnosed glioblastoma patients with recurrent glioblastoma patients, there appears to be no difference in clinical outcomes after imatinib plus RT treatment.^163^

**Table 1:** RTK signalling targeted therapy in GBM clinical trials

Drug	Target	Phase	Indication	No. Patients	Treatment	mOS (weeks)	mPFS (weeks)	PFS6 (%)	PFS12 (%)	Best response to treatment				Ref
										PD	SD	PR	CR	
Imatinib	KIT, PDGFR, BCR-ABL	II	Recurrent	231	Imatinib + Hydroxyurea	26	5.6	10.6		142	45	7	1	^160^
		II	Recurrent	33	Imatinib + Hydroxyurea	48.9	14.4	27		16	14	2	1	^158^
		I/II	Recurrent	35	Imatinib			3			6	2	0	^156^
		III	Recurrent	120	Imatinib + Hydroxyurea	21	6	5	2	93				^161^
		I	Recurrent	34	Imatinib + Hydroxyurea + Vatalanib	48	12	25						^159^
		I	GBM (unspecified)	53	Imatinib + TMZ	45.1	26.6	52.8						^162^
			GBM (unspecified)	51 (19 primary, 32 recurrent)	Imatinib + RT	21.2; 28.3	12.2; 9.1							^163^
		II	Recurrent	50	Imatinib	25.7	7.8	16		13	3	8		^157^
Sorafenib	VEGFR, PDGFR, Raf kinase, KIT, FLT3	II	Newly diagnosed	47	Sorafenib + RT/TMZ	52.2	26.1		16	13	25	6		^167^
		II	Recurrent	32	Sorafenib + TMZ	41.5	6.4	9.4		16	15	1		^168^
		I/II	Recurrent	49 (no prior Bevacizumab); 46 (prior Bevacizumab)	Sorafenib + Temsirolimus	28.7; 17	11.7; 8.3	16.3; 6.8		9; 20	29; 20	4; 1		^169^
		I/II	Recurrent	18	Sorafenib + Temsirolimus		8	0				2		^170^
		I/II	Recurrent	12 (no prior Bevacizumab); 40 (prior Bevacizumab)	Sorafenib + Everolimus			8.33 (PFS6); 12.9 (PFS3)						NCT01434602
		II	Recurrent	54	Sorafenib + Bevacizumab	24.4	12.6	20.4			34	10		^171^
Sunitinib	VEGFR, PDGFR, KIT, FLT3	II	Recurrent	16	Sunitinib	54.8	6.1	12.5			31	0	0	^175^
		II	GBM (Unspecified)	12	Sunitinib + RT	12.8	7.7			11	1	0	0	^172^
		II	Recurrent	32 (no prior Bevacizumab); 31 (prior Bevacizumab)	Sunitinib	40.9; 19		10.4; 0				3; 0		^174^
		II	Recurrent	40	Sunitinib	40	9.57	12.5						^173^
Dasatinib	BCR-ABL, Src, KIT, PDGFR	II	Recurrent	29	Dasatinib	38.7	7.8	6.9				0	0	^192^
		I/II	Recurrent	83	Dasatinib + Bevacizumab	25.2	13.9	28.9			48			^193^
		I/II	Recurrent	26	Dasatinib + CCNU	22.1	4.7	7.7			6	1		^191^
Pazopanib	PDGFR, VEGFR, KIT, FGFR	II	Recurrent	35	Pazopanib	35	12	3		11	21	2		^176^
Vandetanib	EGFR, VEGFR, RET	II	Newly diagnosed	69	Vandetanib + RT/TMZ	72.2	33.5	0.58		8	27	9	4	^179^
		II	Recurrent	32	Vandetanib	27.4	5.7	6.5					1	^178^
		I	Recurrent	19	Vandetanib + Sirolimus	33.5	9.1	18.2				2		^177^
Regorafenib	VEGFR, PDGFR, FGFR, Raf, RET, KIT	II	Recurrent	59	Regorafenib	32.2	8							^181^
Cabozantinib	VEGFR, ROS, TIE2, c-Met, KIT, TRK2, RET	II	Recurrent	117 (no prior antiangiogenic therapy); 58 (prior antiangiogenic therapy)	Cabozantinib	45.2; 20	16.1; 10	27.8; 8.5		14; 16	79; 27	17; 2		^183,184^
Ponatinib	BCR-ABL, PDGFR, VEGFR, FGFR, Src, FLT3, KIT	II	Recurrent	14 (prior Bevacizumab)	Ponatinib	14	4	0			2	0	0	^185^
Nintedanib	VEGFR, PDGFR, FGFR, MDR1, BCRP	II	Recurrent	12 (no prior Bevacizumab); 10 (prior Bevacizumab)	Nintedanib	30; 11.3	4; 4	0; 0			4; 1	0	0	^187^
		II	Recurrent	13 (no prior Bevacizumab); 12 (prior Bevacizumab)	Nintedanib	35.2; 5.7	4	0; 8.3		11; 11	3; 1	0		^186^
Tivozanib	VEGFR, PDGFR, FGFR, KIT, RET	II	Recurrent	10 (no prior Bevacizumab)	Tivozanib	35.2	10	10		4	4	1	1	^188^
Anlotinib	VEGFR, PDGFR, FGFR		Recurrent	20	Anlotinib + TMZ	51.8	26.5	55		1	5	13	1	^190^
			Recurrent	5	Anlotinib + RT				60			3	2	^189^
Pexidartinib	CSF1R, KIT, FLT3	II	Recurrent	37	Pexidartinib	40.1		8.8		35		0	0	^195^
		1b/II	Newly diagnosed	53	Pexidartinib + RT/TMZ	58.3	29.1							NCT01790503
Gefitinib	EGFR	I/II	Newly diagnosed	147	Gefitinib + RT	50	21.3	40						^197^
		II	Newly diagnosed	96	Gefitinib + RT				16.7					^198^
		II	Recurrent	53	Gefitinib	39.4	8.1	13		51				^199^
		II	Recurrent	16	Gefitinib	24.6	8.4	14.3	12.5		3			^200^
		II	Recurrent	38	Gefitinib + Cediranib	31.3	15.7			37		8		^201^
		I/II	Recurrent	22	Gefitinib + Everolimus	24.9	11.3			11	8	3		^202^
Erlotinib	EGFR	I/II	Newly diagnosed	97	Erlotinib + RT/TMZ	66.6	31.3		61					^203^
		II	Newly diagnosed	65	Erlotinib + RT/TMZ	84	35.7							^204^
		II	Newly diagnosed	46	Erlotinib + Bevacizumab + RT/TMZ	57.4	40				28	12	4	^206^
		II	Newly diagnosed	59	Erlotinib + Bevacizumab + RT/TMZ	86.1	58.7							^205^
		I	Recurrent	21	Erlotinib						1		1	^207^
		II	GBM	42 recurrent; 44 newly diagnosed	Erlotinib	26.1; 60.9	8.7;	3;		26;	3;			^208^
		II	Recurrent	48	Erlotinib	42.2		20		26	16	3	1	^209^
		II	Recurrent	54	Erlotinib	33.5	7.83	11.4			9	2		^210^
		II	Recurrent	43	Erlotinib + Carboplatin	30	9	14			20	1		^211^
		II	Recurrent	56	Erlotinib + Sorafenib	24.8	10.9	14		70				^212^
		II	Recurrent	32	Erlotinib + Sirolimus	33.8	6.9	3.1						^213^
		II	Recurrent	24	Erlotinib + Bevacizumab	44.6	18	29.2		2	10	11	1	^215^
		I/II	Recurrent	42	Erlotinib + Temsirolimus		8	13			12			^214^
Dacomitinib	EGFR	II	Recurrent	30 (EGFRamp/ EGFRvIII-); 19 (EGFRamp/ EGFRvIII + )	Dacomitinib	33.9; 29.1	11.7; 11.3	13.3; 5.9		17; 13	8; 4	1; 1	1; 0	^218^
		II	Recurrent	30	Dacomitinib	43	8.9	17			5		1	^219^
Osimertinib	EGFR	R	Recurrent	15	Osimertinib + Bevacizumab	39.2	22.2	46.7			10	1	1	^222^
Depatux-M	EGFR/ EGFRvIII	II	Recurrent	88; 86	Depatux-M + TMZ; Depatux-M	41.8; 34.4	11.7; 8.3			23; 28	17; 10	5; 2		^250^
		III	Newly diagnosed	323	Depatux-M + RT/TMZ	82.2	34.8							^251^
Axitinib	VEGFR	II	Recurrent	50	Axitinib	29	12.4	26		24	12	11	3	^231^
		II	Recurrent	27	Axitinib	18	10.7	18.5		14	7	5	1	^232^
Cediranib	VEGFR	III	Recurrent	131 Cedarinib; 129 Cedarinib + Lomustine	Cedarinib + Lomustine	34.8; 40.1	13.1; 17.9			10; 19	76; 67	17; 19	1; 2	^233^
Apatinib	VEGFR	II	Recurrent	20	Apatinib + TMZ	39.2	26.1			2	9	8	1	^234^
		R	Recurrent	19	Apatinib	45.2	22.2	38.9	16.7	5	4	10		^235^
Olaratumab	PDGFRα	II	Recurrent	40	Olaratumab	34.3		7.5				1		NCT00895180
Tovetumab	PDGFRα	II	Recurrent	56	Tovetumab	42.2	6.1	15.4			23	0		^253^
Capmatinib	c-MET	I/II	Recurrent	10	Capmatinib						3	0	0	^240^
Crizotinib	c-MET	I	Newly diagnosed	36	Crizotinib + RT/TMZ	98.3	46.5			2	13	5	1	^241^
Onartuzumab	c-Met	II	Recurrent	59	Onartuzumab + Bevacizuamb	38.3	17	33.9				11	1	^254^
Buparlisib	Pan-PI3K	II	Recurrent	17 buparlisib + carboplatin; 18 buparlisib + lomustine	Buparlisib + Chemotherapy		6.1; 5.7			13; 14	3; 2	1; 0	0; 0	^257^
		II	Recurrent	15 buparlisib + surgery; 50 buparlisib	Buparlisib	77.9; 42.6	7.8; 7.4	26.7; 8		9; 27	6; 21	0; 0	0; 0	^258^
		II	Recurrent	17 prior bevacizumab; 50 bevacizumab-naïve	Buparlisib + Bevacizumab	28.7; 47	9.3; 23.1	15.8; 44		8; 14	7; 18	0; 12	2; 6	^259^
Sonolisib	Pan-PI3K	II	Recurrent	33	Sonolisib			17			8	1		^260^
Everolimus	mTORC1	II	Newly diagnosed	100	Everolimus + RT/TMZ	68.7	27.8							^267^
		II	Newly diagnosed	88	Everolimus + RT/TMZ	71.8	35.7							^268^
		II	Newly diagnosed	68	Everolimus + Bevacizumab + RT/TMZ	60.5	49.2	73	42			29		^269^
Temsirolimus	mTORC1	II	Newly diagnosed	56	Temsirolimus + RT	64.4	23.5							^270^
		II	Recurrent	41	Temsirolimus		9	2.4			20	2		^272^
		II	Recurrent	65	Temsirolimus	19.1	10	7.8						^271^
		II	Recurrent	13	Temsirolimus + Bevacizumab	15	8				2			^273^
Enzastaurin	PKCβ	II	Newly diagnosed	66	Enzastaurin + RT/TMZ	74	36	65						^289^
		II	Newly diagnosed	57	Enzastaurin + RT	65.3	28.7	53.6	14.9		31	3	1	^288^
		III	Recurrent	266	Enzastaurin	28.7	6.5	11.1		72	67	5		^290^
		II	Recurrent	40	Enzastaurin + Bevacizumab	32.6	8.7	21			20	8		^291^
Dabrafenib/ Trametinib	BRAF/ MEK	II	Recurrent	31	Dabrafenib + Trametinib	59.6	12.2			14	6	8	2	^293^
Vemurafenib	BRAF	II	Recurrent (BRAF V600-mutant malignant diffuse gliomas)	11 (6 GBM)	Vemurafenib	51.8	23.1			3	5	1	0	^294^

*ABL* abelson murine leukaemia viral oncogene homologue 1, *BCR* breakpoint cluster region, *BCRP* breast cancer resistance protein, *BET* bromodomain and extraterminal-containing protein family, *CCNU* cyclonexyl-chloroethyl-nitrosourea, *CR* complete response, *CSF1R* colony stimulating factor 1 receptor, *Depatux-M* Depatuxizumab mafodotin, *DNMT* DNA methyl transferase, *EGFR* epidermal growth factor receptor, *FGFR* fibroblast growth factor receptor, *FLT3* FMS-like tyrosine kinase 3, *KIT* receptor tyrosine kinase, *GBM* glioblastoma, *IGF1R* insulin-like growth factor 1 receptor, *MDR1* multi-drug resistance 1, *mOS* median overall survival, *mPFS* median progression free survival, *PD* progressive disease, *PDGFR* platelet-derived growth factor, *PFS6* progression free survival at 6 months, *PFS12* progression free survival at 12 months, *PR* partial response, *R* retrospective, *RT* radiotherapy, *SD* stable disease, *TMZ* temozolomide, *VEGFR* vascular endothelial growth factor

Various small molecule inhibitors with multi-targeting RTK activity have been developed, most of which mainly inhibit VEGFR, PDGFR, or both. Sorafenib, which targets VEGF, PDGFR, Raf kinase, receptor tyrosine kinase (KIT), and FMS-like tyrosine kinase 3 (FLT3), is approved for treating advanced hepatocellular carcinoma and advanced renal cell carcinoma.^166^ Neither primary glioblastoma nor recurrent glioblastoma patients had improved clinical outcomes when treated with sorafenib and TMZ in phase II clinical trials.^167,168^ Sorafenib has also been trialled as a combination therapy with temsirolimus, an mTOR inhibitor, in recurrent glioblastoma patients who had been previously treated with bevacizumab. In a phase I/II trial, recurrent glioblastoma patients who were anti-VEGF therapy-naïve had greater response and survival rates compared with patients who had previously failed bevacizumab therapy (PFS at 6 months of 16.3% vs 6.8%, respectively).^169^ However, in another study of bevacizumab therapy-naïve recurrent glioblastoma patients, the median PFS time was only 8 weeks after sorafenib and temsirolimus treatment, and no patients remained progression-free at 6 months.^170^ Furthermore, both studies found this combination approach to have high rates of grade 3 adverse events; therefore, the ineffective and toxic combination of sorafenib and temsirolimus has not been explored further. A combination of sorafenib and another mTOR inhibitor, everolimus, appears to have fewer toxic side effects in recurrent glioblastoma patients, however, this treatment regime still fails to improve patient survival (NCT01434602). Recurrent glioblastoma patients treated with a combination of sorafenib and evacizumab also experienced high levels of toxicity at the recommended doses for clinical activity, leading to failure to continue treatment and no improvement in clinical outcomes compared with historical bevacizumab-treated patients.^171^

Other multi-targeting RTK inhibitors that have been considered in glioblastoma treatment include sunitinib,^172–175^ pazopanib,^176^ vandetanib,^177–179^ regorafenib,^180–182^ cabozantinib,^183,184^ ponatinib,^185^ nintedanib,^186,187^ tivozanib,^188^ and anlotinib.^189,190^ These drugs have been trialled mostly in recurrent glioblastoma patients, either as single-agent therapy or in combination therapy with RT/TMZ, but with limited success (Table 1). As monotherapy agents, treatment with sunitinib, pazopanib, vandetanib, ponatinib, nintedanib, and tivozanib resulted in a wide range of OS (14–55 weeks) in recurrent glioblastoma patients in phase II clinical trials. However, none of these drug candidates met the primary endpoint for PFS at 6 months (<15% of patients).^173,174,176,178,185–187^ The REGOMA clinical trial reported significantly improved OS rates in recurrent glioblastoma patients receiving regorafenib compared with those receiving lomustine,^181^ a standard second-line therapy for recurrent glioblastoma. However, the median OS of 32 weeks in the REGOMA trial is comparable to the many other multi-targeted RTK inhibitors assessed in clinical trials. Therefore, regorafenib does not appear to be superior to other RTK inhibitors nor does it improve the average survival time of recurrent glioblastoma patients. However, some individual case reports have documented favourable radiological responses in patients treated with regorafenib as a second or third-line therapy.^180,182^

Several RTK inhibitors have been assessed in recurrent glioblastoma patients who failed prior anti-VEGF therapy (bevacizumab) and in those who were naïve to bevacizumab treatment. Recurrent glioblastoma patients who do not respond to bevacizumab treatment have notoriously worse survival rates than those who do respond. As expected, the recurrent glioblastoma patients who had previous bevacizumab therapy had very poor outcomes, with only 0–10% of patients remaining progression-free at 6 months when treated with sunitinib,^174^ cabozantinib,^183^ or nintedanib.^186,187^

Anlotinib is another RTK inhibitor yet to enter clinical trials for glioblastoma. However, a retrospective study of glioblastoma patients treated with anlotinib and TMZ reported an OS of 12 months and an impressive PFS time of 6 months.^190^ Furthermore, a preliminary study of five glioblastoma patients treated with anlotinib and RT found a 100% radiographic response rate, with three of the five patients remaining progression-free at 12 months.^189^ These outcomes are an improvement from those typically observed in recurrent glioblastoma patients treated with bevacizumab. Therefore, clinical trials should be implemented to fully examine the potential therapeutic benefits of anlotinib in recurrent glioblastoma patients.

Only two FDA-approved small molecule inhibitors, dasatinib and pexidartinib, target proteins other than VEGFR. Dasatinib targets the active form of BCR-ABL, as well as Src, c-KIT, and PDGFR. Recurrent glioblastoma patients treated with dasatinib alone or in combination with CCNU (cyclonexyl-chloroethyl-nitrosourea) did not demonstrate favourable outcomes, with a median PFS of less than two months.^191,192^ The combination of dasatinib and bevacizumab resulted in higher PFS time of 3.2 months in recurrent glioblastoma patients, although these outcomes are not superior to bevacizumab treatment alone.^193^ Pexidartinib is an inhibitor of c-KIT, FLT3, and colony-stimulating factor 1 (CSF-1), a receptor that belongs to the PDGFR family. CSF-1 is responsible for PI3K/AKT signalling to promote survival and proliferation of tumour cells and tumour-associated macrophages.^194^ Pexidartinib is ineffective as a single agent therapy in recurrent glioblastoma patients, with 95% of patients demonstrating disease progression in a phase II clinical trial.^195^ Furthermore, newly diagnosed glioblastoma patients treated with pexidartinib in combination with RT/TMZ had an OS and PFS of 58 weeks and 29 weeks, respectively, demonstrating no improvement, compared with traditional RT/TMZ treatment (NCT01790503).

### Small molecule EGFR inhibitors

Considering the large proportion of glioblastoma patients with EGFR mutations, small molecule inhibitors targeting EGFR have been at the forefront of clinical trials. Despite the prominent role that EGFR plays in glioblastoma development and growth, therapeutic strategies targeted to the receptor have not been successful.^45^ Gefitinib was the second FDA-approved small molecule inhibitor and the first FDA-approved EGFR inhibitor, originally for treating non-small cell lung cancer patients harbouring EGFR mutations.^196^ In phase II clinical trials for newly diagnosed and recurrent glioblastoma patients, gefitinib offered no clinical benefit, with a median OS of 5–12 months.^197–202^

Erlotinib is another first-generation EGFR inhibitor that, like gefitinib, reversibly inhibits ATP binding to EGFR and blocks phosphorylation and signal transduction. Erlotinib in combination with traditional RT/TMZ has been trialled in phase II studies in primary glioblastoma patients. A phase I/II trial of 97 primary glioblastoma patients treated with erlotinib plus RT/TMZ found no improvements in OS or PFS compared with historical outcomes of RT/TMZ treatment alone.^203^ In contrast, another phase II trial reported by Prados and colleagues showed that primary glioblastoma patients treated with erlotinib plus RT/TMZ had significantly improved OS (19.3 months versus 14.1 months in historical controls) and PFS (8.2 months versus 4.9 months in historical controls).^204^ Notably, the reported PFS used for a historical control in this study is substantially lower than what has been previously reported for RT/TMZ treatment,^2,203^ which likely affected the statistical significance of the reported outcomes.

Building on these results, studies have investigated the effects of a treatment approach for primary glioblastoma patients including RT/TMZ in combination with erlotinib plus bevacizumab, however, the efficacy of this treatment regime is also controversial. While two individual trials found no significant improvement in OS, compared with previously published data,^205,206^ one of these studies found a significant improvement in median PFS (13.5 months versus 8.6 months in historical controls).^205^ Although Prados and colleagues found significantly improved OS and PFS in primary glioblastoma patients treated with erlotinib plus RT/TMZ, as described above,^204^ Clarke and colleagues^205^ found only a significant improvement in PFS, but not in OS, for primary glioblastoma patients treated with RT/TMZ, erlotinib and bevacizumab, when compared with the outcomes of the trial by Prados et al. as a historical control. Once again, the inconsistent use of historical control populations used across these clinical trials makes it difficult to draw conclusions on the agent-specific efficacy of these treatment regimes. Thus, the combination of erlotinib plus RT/TMZ, with or without the addition of bevacizumab, is unlikely to have any substantial long-term benefit in glioblastoma patients.

Regarding recurrent glioblastoma, clinical trials have considered erlotinib alone^207–210^ or a combination of erlotinib and carboplatin (chemotherapy),^211^ erlotinib and sorafenib (Raf inhibitor),^212^ or erlotinib and mTOR inhibitors (sirolimus and temsirolimus).^213,214^ These trials have not resulted in positive outcomes, with the average PFS of patients ranging from 6–11 weeks. However, one study found that the combination of erlotinib and bevacizumab resulted in a PFS time of 18 weeks in 24 recurrent glioblastoma patients, with 50% of patients showing radiographic response and nearly 30% of patients remaining progression-free at 6 months.^215^ An even more impressive prospective study has shown that molecular profiling of EGFR and VEGF status in recurrent glioblastoma patients may be a more effective strategy for disease management. D’Alessandris and colleagues^216^ showed a 100% radiographic response rate with erlotinib plus bevacizumab treatment in recurrent glioblastoma patients pre-screened for high EGFR and VEGF expression. In this subset of patients, the median OS and PFS were 18 months and 10.5 months, respectively; a remarkable improvement from otherwise failed clinical trials in recurrent glioblastoma patients treated with erlotinib. Considering this preliminary study only included 10 patients, of which only four received this combination treatment, this profiling strategy should be implemented in larger clinical trials.

Later-generation EGFR inhibitors, dacomitinib and osimertinib, were developed as irreversible inhibitors of EGFR with activity against EGFRvIII. Dacomitinib is a second-generation EGFR inhibitor that has been tested in a phase II clinical trial of recurrent glioblastoma patients with EGFR-amplified tumours either with or without EGFRvIII expression. Despite the anti-EGFRvIII activity observed in pre-clinical glioblastoma models,^217^ dacomitinib-treated recurrent glioblastoma patients with EGFR amplification/EGFRvIII expression had poorer survival outcomes compared with dacomitinib-treated recurrent glioblastoma patients with EGFRvIII negative tumours.^218^ Likewise, another phase II trial found that EGFRvIII expression in dacomitinib-treated recurrent glioblastoma patients was not associated with any clinical benefit.^219^

Osimertinib is a third-generation EGFR inhibitor demonstrated to have superior potency for inhibiting EGFRvIII activity.^220,221^ Osimertinib has not been explored in clinical trials of glioblastoma patients, however, a retrospective study of 15 recurrent glioblastoma patients treated with osimertinib and bevacizumab reported a 6-month PFS rate of 46%.^222^ A case report of a woman diagnosed with glioblastoma who had progressive disease after attempts at RT/TMZ, surgery, and bevacizumab was given osimertinib in an off-label, off-protocol manner. After one month of daily osimertinib treatment, the patient showed a near-complete response in one of the two tumour masses. The responsive tumour harboured EGFR amplification and was EGFRvIII-negative, whereas the progressive tumour was EGFRvIII-positive.^223^ Although there are several possible explanations for this observation, it is clear that small molecule EGFR inhibitors have limited efficacy in glioblastoma patients due to the complex nature of EGFR activity in glioblastoma tumours.

As a more novel approach to exploiting EGFR activity in glioblastoma therapy, future research endeavours could focus on targeting the androgen receptor (AR) in combination with anti-EGFR treatments. While the AR typically functions as a steroid hormone-activated transcription factor,^224^ it has been shown in glioblastoma cells that AR activation may also occur in a ligand-independent manner via EGFR signalling.^225^ AR RNA and protein expression may be induced in as much as 93 and 56% of glioblastoma patients, respectively.^226^ Thus, the functional significance of AR and its relation to EGFR signalling is of growing interest. AR expression has been shown to be strongly correlated with EGFR expression in glioblastoma patients.^225^ Furthermore, although there appears to be no differences in AR expression between male and female glioblastoma patients, AR expression may vary within the tumour regions, demonstrating higher protein expression levels in the tumour periphery and peritumoural regions,^227,228^ and stronger protein localisation in CD133 positive cells, indicating a potential functional significance in glioma stem cells.^228^ Enzalutamide, an AR inhibitor that is FDA-approved for prostate cancer, was shown to decrease the density of cancer stem cell populations and improved survival by 50% in a glioblastoma orthotopic mouse model.^228^ Interestingly, co-treatment of enzalutamide and afatinib (ErbB family inhibitor) had increased efficacy in reducing survival of EGFR-expressing glioblastoma cells, however, a combination of enzalutamide and erlotinib or cetuximab (EGFR selective inhibitors) did not display additive effects on EGFR expressing cells.^225^ Despite the obvious benefits of enzalutamide as a novel treatment approach, including increased AR expression in many high-grade gliomas, high BBB penetrance,^229^ and the ability to detect AR-positive gliomas in real time with a 16β-18F-fluoro-5α-dihydrotestosterone ([18F]-FDHT) positron emission tomography (PET) tracer,^230^ the pre-clinical data in support of enzalutamide for having a significant improvement in glioblastoma survival outcomes, either alone or in combination with EGFR inhibitors, is lacking. This combination strategy has yet to be tested in vivo, and future research should consider the probability that AR activation may still occur via other RTK-related signalling mechanisms. As an emerging therapeutic strategy, targeting the RTK/AR axis is one to be explored in greater depths before any conclusions can be drawn on its benefit in future glioblastoma clinical trials.

### Small molecule VEGFR/PDGFR/FGFR/c-MET inhibitors

As discussed above, most of the RTKs that have been approved or are currently in development are non-selective multi-TKIs. Few of the developed small molecule inhibitors have selectivity for only VEGFR, PDGFR, or FGFR. Axitinib selectively blocks VEGFR-1, VEGFR-2, and VEGFR-3. PFS rates in phase II clinical studies in recurrent glioblastoma patients receiving axitinib are favourable compared with those of other multi-RTK inhibitors such as sunitinib and pazopanib, but are not superior to historical bevacizumab outcomes.^231,232^ Cedarinib is another pan-VEGFR inhibitor and is one of few small molecule inhibitors to make it to phase III clinical trial in glioblastoma. However, cedarinib treatment did not meet the PFS endpoint either as a monotherapy or in combination with lomustine.^233^ Apatinib selectively inhibits VEGFR-2 and results in an average PFS time of 24 weeks in recurrent glioblastoma patients.^234,235^ Although there are few clinical trials investigating apatinib in recurrent patients, three independent case reports of glioblastoma patients reported stable disease, improved symptoms, and prolonged survival with apatinib treatment following RT/TMZ-induced pseudoprogression.^236–238^ Apatinib is formulated as an oral tablet, which is a more convenient and cheaper option than intravenous injection of bevacizumab, and may provide similar clinical benefit to progressive glioblastoma patients.^236^

While there are some selective inhibitors against PDGFR (avapritinib and ripretinib) and FGFR (erdafitinib, pemigatinib, and infigratinib), there is insufficient clinical data on their use in glioblastoma patients. A recent study reported limited efficacy of infigratinib in patients with recurrent gliomas, which included glioblastoma as well as anaplastic astrocytomas and undefined gliomas.^239^ Currently ongoing clinical trials are recruiting recurrent glioblastoma patients harbouring FGFR mutations to further evaluate the efficacy of infigratinib (NCT04424966) and pemigatinib (NCT05267106).

Capmatinib is one of the few selective c-Met inhibitors to enter clinical trials for glioblastoma, but had little clinical activity in PTEN-deficient recurrent glioblastoma patients.^240^ Clinical trials of the combination of capmatinib and bevacizumab are currently underway in glioblastoma patients (NCT02386826). Crizotinib is another selective c-MET inhibitor that had encouraging results in phase I clinical trials in newly diagnosed glioblastoma patients. Patients treated with crizotinib plus RT/TMZ had an OS of 22.6 months and PFS of 10.7 months, which is higher than the expected outcomes for RT/TMZ alone (14.6 months and 6.9 months, respectively).^241^ Therefore, larger phase II clinical trials for this combination are warranted. Although it is known that c-Met crosstalk with EGFR, particularly EGFRvIII, drives glioblastoma pathogenesis and treatment resistance, clinical trials have yet to investigate the combination of EGFR inhibitors with c-Met inhibitors. A pre-clinical study has shown potent synergistic effects in mice bearing patient-derived glioblastoma tumours treated with a combination of crizotinib plus erlotinib.^242^ Therefore, this combination may be a worthy therapeutic strategy to explore in the clinic to delay treatment resistance and increase survival in EGFRvIII^+^/c-Met^+^ glioblastoma patients.

### Anti-RTK antibodies

Numerous EGFR-targeting antibodies have been clinically approved for the treatment of a range of cancers.^243^ Moreover, the majority of antibody trials in glioblastoma have used antibodies directed to EGFR. However, many of these have been used to treat glioblastoma patients “off-label” and rigorous phase III trials are lacking.^244^ Also, none of the reported studies selected patients who might best respond to EGFR antibody therapy. Importantly we recently showed that only one EGFR antibody, panitumumab, was able to neutralise both the wild-type EGFR and EGFRvIII in cell and animal models.^245^ Given these shortcomings, naked EGFR-specific antibodies have not progressed as therapies.

There was initial concern that anti-EGFR antibodies would not cross the BBB effectively. The use of antibodies with payloads, which are more effective cell killers, is one method of lowering the amount of antibody that needs to cross the BBB to have a clinical effect. Early trials have shown that antibody-drug conjugates (ADCs) directed to targets found in secondary brain cancer have therapeutic efficacy.^246^ For example, trastuzumab emtansine, an anti-HER2 (also known as ERBB2, a member of the EGFR RTK family) ADC, has shown promising anti-tumour activity in breast cancer metastasis to the brain (recently reviewed ref. ^246^).

As discussed above, EGFRvIII is a cancer-specific target. Antibodies that target this receptor could potentially be used to deliver payloads to glioblastoma, although it would be limited to the 30% of patients that express this receptor. mAb 806 is an unique EGFR-specific antibody that was developed to target EGFRvIII but also can bind to autoactive forms of the EGFR.^247–249^ Thus, it binds EGFRvIII, overexpressed EGFR, and EGFR with point mutations, which are collectively present in approximately 60% of glioblastoma patients.^32^ mAb 806 was developed into Depatux-M, a humanised mAb 806 linked to the microtubule inhibitor monomethylauristatin F.^246^ A phase II trial of Depatux-M in relapsed glioblastoma patients showed exciting clinical activity.^250^ However, a phase III trial with Depatux-M in newly diagnosed glioblastoma patients showed no clinical efficacy at all (Table 1).^251^ One possible explanation is that the BBB is more disrupted in relapsed disease and, therefore, more Depatux-M reached the tumour site in these patients.^252^ Optimisation of payloads and linkers are required to progress this therapeutic strategy and to hopefully develop effective anti-EGFR antibodies for the treatment of glioblastoma.^246^

Bevacizumab is by far the most well-studied monoclonal antibody targeting VEGFR, and has been extensively studied in glioblastoma. As previously mentioned, it is an approved treatment for recurrent glioblastoma patients and will not be discussed in further detail here. Very few other monoclonal antibodies targeting VEGFR, PDGFR, FGFR or c-Met have been developed, let alone trialled in glioblastoma. Two anti-PDGFRα monoclonal antibodies, olaratumab (NCT00895180) and tovetumab,^253^ have been trialled in recurrent glioblastoma patients, but neither showed significant clinical activity (Table 1). Although antibodies targeting FGFR2 (bemarituzumab, NCT03694522) and FGFR3 (vofatamab, NCT03123055; LY3076226, NCT02529553; MFGR1877S, NCT01363024) are in clinical trials for other cancers, these have yet to be considered for glioblastoma. The presence of FGFR3/TACC fusion proteins in some glioblastoma patients suggests that FGFR3 antibodies could be considered as a therapeutic option for select patients with this alteration. The only c-Met monoclonal inhibitor that has been studied in glioblastoma is onartuzumab, which was given to recurrent glioblastoma patients either as a monotherapy or in combination with bevacizumab (Table 1). Although there was no difference in survival outcomes between the two treatment groups overall, subgroup analyses suggest that patients with high *HGF* expression had significantly improved PFS when treated with onartuzumab plus bevacizumab.^254^

### Small molecule PI3K/AKT/mTOR inhibitors

As discussed above, the PI3K/AKT/mTOR pathway is a significant interconnected signalling cascade that regulates cell proliferation, metabolic regulation, cell cycle progression, and angiogenesis. Although this network is complex, it is a master regulator of tumour development and progression, therefore, there have been extensive efforts to develop targeted therapies against PI3K/AKT/mTOR signalling. Since AKT mutations have not been observed in glioblastoma,^255^ the emphasis has been placed on PI3K and mTOR inhibitors.

PI3K inhibitors are classified as pan-PI3K inhibitors, which are ATP-competitive inhibitors that target all four isoforms, isoform-selective inhibitors, or dual PI3K/mTOR inhibitors. Buparlisib (BKM120), a pan-PI3K inhibitor, in combination with RT/TMZ has been evaluated in phase I clinical trials in newly diagnosed glioblastoma patients, but did not progress further due to significant toxicities resulting in treatment discontinuation.^256^ In phase II clinical trials of recurrent glioblastoma patients, there were no clinical responses in patients treated with buparlisib and chemotherapy^257^ or in patients treated with buparlisib alone or surrounding surgical resection.^258^ Bevacizumab-naïve recurrent glioblastoma patients treated with buparlisib and bevacizumab have better outcomes and clinical response compared with patients who were previously treated with bevacizumab, but this is likely due to the effects of bevacizumab rather than those of buparlisib.^259^ Sonolisib (PX-866), another pan-PI3K inhibitor, also failed to meet primary endpoints in recurrent glioblastoma patients.^260^ Two other pan-PI3K inhibitors, pilaralisib (XL147, NCT01240460) and pictisilib (GDC-0941, NCT02430363), are in phase II clinical trials for recurrent glioblastoma, however, they may have limited BBB penetration capacity.^261,262^

Isoform-specific PI3K inhibitors may have reduced off-target effects and toxicities. Inhibitors that target the PI3K isoforms p110α, p110β and p110δ are the most relevant since these isoforms are differentially expressed in glioblastoma.^85^ While there are some p110α/p110β and several p110δ inhibitors on the market, they have not been evaluated in the clinical setting for glioblastoma. p110β is important for reducing glioblastoma growth in pre-clinical models.^85^ This may explain, in part, why buparlisib failed as a treatment for glioblastoma, because a recent study has shown that buparlisib failed to inhibit p110β in glioblastoma mouse models.^263^ Despite the clear importance of p110β in tumourigenesis, there is currently only one specific p110β inhibitor (GSK2636771). The results of the first clinical trial conducted on patients with PTEN-deficient advanced solid tumours to evaluate the safety and efficacy of GSK2636771 have yet to be described (NCT01458067).

mTOR inhibitors have been developed since the 1990s, beginning with the discovery of rapamycin isolated from *Streptomyces hygroscopicus* in 1972. Analogues of rapamycin (rapalogs), including sirolimus, temsirolimus, everolimus, and ridaforolimus, are first-generation mTOR inhibitors that specifically inhibit mTORC1. Phase I studies of everolimus with RT/TMZ treatment in newly diagnosed and recurrent glioblastoma patients was well-tolerated.^264–266^ However, phase II clinical trials investigating everolimus combined with RT/TMZ in newly diagnosed glioblastoma patients showed no improvement in PFS.^267,268^ A clinical study of standard RT/TMZ treatment followed by maintenance therapy with concurrent daily everolimus and fortnightly bevacizumab, resulted in favourable PFS outcomes compared with RT/TMZ treatment alone, but the treatment regime was not superior to previous studies of RT/TMZ plus maintenance bevacizumab.^269^ In a phase II trial for temsirolimus plus RT there was no clinical benefit to newly diagnosed glioblastoma patients compared with RT/TMZ treatment, however, a subset of patients with phosphorylation of mTOR at serine 2448 had an association with clinical benefit from temsirolimus.^270^ Similarly, temsirolimus treatment in recurrent glioblastoma patients did not improve survival outcomes;^271–273^ although one study showed that glioblastoma patients with phosphorylated AKT were associated with temsirolimus response.^271^ Other unreported clinical trials have investigated sirolimus (NCT00047073), temsirolimus (NCT01051557) and ridaforolimus (NCT00087451) in glioblastoma patients. These data show that collectively, first generation mTORC1 inhibitors do not benefit glioblastoma patients, likely due to feedback loops in the PI3K/AKT/mTOR signalling network, mainly from mTORC2 and AKT activation, which are not blocked by these rapalogs.

Second-generation mTOR inhibitors overcome this problem and are ATP-competitive mTOR inhibitors of both mTORC1 and mTORC2. Vistusertib is a more effective mTOR inhibitor that demonstrates reduced phosphorylation of AKT in vivo.^274,275^ In pre-clinical work, vistusertib has been shown to enhance radiosensitivity in glioblastoma stem-like cells.^276^ A phase I dose determination trial for vistusertib in progressive glioblastoma patients reported a dose of 125 mg in combination with TMZ was well-tolerated with mild adverse events and a PFS rate of 26.6%.^277^ Other ATP-competitive mTOR inhibitors include PP242^278–280^ and torin1/2,^281^ which are effective at reducing mTOR/AKT signalling in glioblastoma in vitro and in vivo, but these agents have yet to enter clinical trials.

Dual PI3K/mTOR inhibitors that have been explored in pre-clinical glioblastoma models include dactolisib, voxtalisib, and paxalisib. Dactolisib treatment combined with RT/TMZ enhanced anti-tumour activity in vitro and in vivo in pre-clinical models of glioblastoma, compared with RT/TMZ alone.^282^ However, another murine study showed that dactolisib alone resulted in severe side effects and did not demonstrate any survival benefit or glioblastoma tumour inhibition.^283^ Therefore, although there is potential for the combination of RT/TMZ plus dactolisib to result in improved tumour response in glioblastoma, this strategy may not be well-tolerated in patients, similar to previous observations involving combination therapy with mTOR inhibitors. Dactolisib is currently in a phase II clinical trial in glioblastoma patients alongside other PI3K/AKT pathway inhibitors (NCT02430363). Voxtalisib has been examined in combination with TMZ with or without RT in a phase I clinical study of newly diagnosed and recurrent glioblastoma, demonstrating a reasonable safety profile but with limited evidence of anti-tumour activity in glioblastoma patients.^284^ Paxalisib (GDC-0084) is a brain-penetrant PI3K/mTOR inhibitor that potently inhibits AKT phosphorylation and reduces orthotopic tumour growth in vivo.^285,286^ Paxalisib is currently in phase I/II clinical trials for both newly diagnosed and progressive/recurrent glioblastoma in adults (NCT03522298, NCT01547546) and children (NCT05009992) with encouraging preliminary results.^287^

### Small molecule PKC inhibitors

PKC isoforms are variably expressed in different cancer types. Although there is abundant pre-clinical evidence to suggest that all PKC isoforms have oncogenic roles in glioblastoma development, the relevant clinical expression of these isoforms is still unknown. Nonetheless, various PKC inhibitors have been trialled in the glioblastoma setting, most notably enzastaurin, a selective PKCβ inhibitor. For newly diagnosed glioblastoma patients, enzastaurin treatment in combination with RT^288^ or RT/TMZ^289^ resulted in comparable survival outcomes to standard of care. Similarly, a phase III clinical trial in recurrent glioblastoma patients showed no difference in outcomes in patients treated with enzastaurin, compared with those treated with lomustine.^290^ Likewise, enzastaurin in combination with bevacizumab showed similar outcomes to historical bevacizumab monotherapy in recurrent glioblastoma patients.^291^

### Small molecule BRAF/MEK inhibitors

As the second major signalling cascade involved in cancer pathogenesis and one of the most frequently mutated pathways in all human cancers, the BRAF/MEK/ERK pathway has been a major target for clinical therapeutic development, especially in cancers where *BRAF* mutations are common, such as in melanoma and thyroid cancer. Considering the infrequent occurrence of *BRAF* mutations in glioblastoma, there is little clinical data on the efficacy of BRAF inhibitors in glioblastoma specifically, rather, clinical trials may include all types of BRAF V600E-mutant glioma. Dabrafenib, encorafenib, and vemurafenib are FDA-approved small molecule inhibitors of BRAF and are frequently used in combination with MEK inhibitors to treat *BRAF*-mutant melanoma patients. Combinations include dabrafenib/trametinib (Novartis), encorafenib/binimetinib (Array BioPharma), and vemurafenib/cobimetinib (Genentech).

There is only one ongoing clinical trial investigating the efficacy of encorafenib/binimetinib in recurrent BRAF V600E/K-mutated glioma patients (NCT03973918). Encouraging preliminary results from this study have reported complete responses in the two glioblastoma cases enroled in the trial.^292^ Dabrafenib/trametinib is currently in clinical trials for newly diagnosed and recurrent glioma patients with the BRAF V600E mutation (NCT03919071, NCT03593993). Recently, the first published phase II clinical trial for dabrafenib/trametinib treatment in BRAF V600E-mutant glioma patients, conducted across 27 institutes, described an overall objective response rate of 32% in glioblastoma patients. Although encouraging, this was much lower than the response rate in low-grade glioma patients (69%). Furthermore, there was no substantial improvement in survival outcomes in patients treated with dabrafenib/trametinib.^293^ Another clinical trial observed similar outcomes in *BRAF*-mutant gliomas treated with vemurafenib as a monotherapy.^294^ In 11 patients with malignant diffuse glioma (six glioblastoma and five anaplastic astrocytoma), the best clinical response was a partial response in one anaplastic astrocytoma patient and stable disease in three glioblastoma patients.^294^ Once again, patients with low-grade gliomas appeared to have the greatest degree of efficacy,^294^ suggesting that BRAF-targeted therapy is unlikely to provide long-term clinical benefit to glioblastoma patients. However, several case reports have described prolonged PFS upwards of 15 months following vemurafenib monotherapy in recurrent cases of *BRAF* V600-mutant glioblastoma,^295,296^ including a complete response in a paediatric glioblastoma patient.^297^ Notably, these patients were negative for *IDH* and *EGFR* mutations, which may explain the positive response.

*EGFR* amplification/mutation and *mTOR* mutations may be significant resistance mechanisms associated with BRAF-targeted therapy, as signalling through the EGFR/PI3K/AKT/mTOR cascade overcomes the block on BRAF/MEK to continue cell proliferation.^116^ This phenomenon should be explored further to identify the specific subsets of *BRAF*-mutant glioblastoma patients that may benefit from BRAF/MEK therapy alone and to clarify whether the addition of EGFR or mTOR inhibitors is required for BRAF-resistant tumours. This triple threat strategy has been explored in colorectal cancer,^298^ but has yet to be considered in the context of glioblastoma or other relevant brain tumours at risk of therapeutic resistance.

## Epigenetic mechanisms in glioblastoma

Epigenetic mechanisms are becoming increasingly relevant in our understanding of glioblastoma pathogenesis and in treatment strategies. Pro-tumoural epigenetic changes include histone modifications, DNA methylation, and chromatin remodelling that inappropriately alter gene expression patterns without modifications to the DNA sequence. Considering that gliomas are typically associated with a low tumour mutational burden (TMB),^299^ epigenetic flexibility likely plays a role in glioblastoma tumour plasticity and contributes to the phenotypically heterogenous population of reversible cell states described above.^35,300^ The importance of phenotypic plasticity in glioblastoma cannot be understated. In support of this, Douglas Hanahan, one of the pioneer authors who first described cancer hallmarks, has recently published a paper describing phenotypic plasticity as an emerging hallmark and epigenetic reprogramming as an enabling characteristic of cancer.^301^ Undoubtedly, epigenetic reprogramming is inherently linked, at least in part, to cellular plasticity and therefore, the signalling pathways discussed thus far. Our understanding of how epigenetic mechanisms regulate oncogenic pathways has inspired the development of targeted therapies against epigenome modulators as a therapeutic strategy for glioblastoma patients.

### DNA methylation

Inappropriate methylation patterns can promote tumour growth. Global hypomethylation patterns encourage oncogene activation and genome instability, and focal hypermethylation at promoter regions can inhibit tumour suppressor genes.^302^ For the latter, DNA methylation takes place on the 5-carbon position on cysteine residues in CpG islands in promoter regions, leading to suppression of gene expression. DNA methyltransferases (DNMTs) are a four-membered protein family responsible for catalysing this reaction. As a well-known prognostic marker, glioblastomas with hypermethylated promoter regions for *O*^6^*-methylguanine DNA methyltransferase* (*MGMT*), a gene responsible for DNA repair of O^6^-methylguanosine, are associated with better response to TMZ and improved survival rates.^2,303^ Furthermore, genome-wide methylation profiling has shown that gliomas with an overall hypermethylated pattern at CpG islands (the CpG island methylator phenotype or G-CIMP) are strongly associated with other prediction markers, including *IDH1* mutation and *MGMT* promoter methylation status. G-CIMP is a better predictor of glioma survival than *MGMT* status alone.^304^ As expected, a large portion of glioblastoma tumours are G-CIMP negative.^305^ Such hypomethylated phenotypes are likely to be directly associated with continued expression of RTK-associated oncogenes. Hypermethylation patterns have also been observed at the *BCL2L11* promoter, the gene responsible for producing the pro-apoptotic protein BIM.^306^ Interestingly, EGFR inhibitor-resistant glioblastoma has decreased BIM expression in vitro and in vivo.^307^ Thus, DNMTs play a critical role in the aberrant methylation patterns that contribute to downregulation of tumour suppressor genes.

RTK signalling plays a role in regulating these epigenetic modulators. As mentioned previously, PI3K signalling and AKT activation inhibits the downstream target GSK-3β. AKT-mediated inactivation of GSK-3β results in reduced expression of DNMT3 and hypomethylation of imprinted DNA regions in stem cells,^308^ providing a potential feed-forward loop that promotes RTK signal transduction.

### Histone modulators

Histone modification, another well-characterised epigenetic mechanism, includes histone methylation, acetylation, phosphorylation, ubiquitination, and ADP-ribosylation.^309^ Histone acetylation and methylation are most relevant to glioblastoma tumourigenesis and are mediated by histone deacetylases (HDACs), histone methyltransferases and epigenetic reader proteins, mainly the bromodomain and extraterminal-containing protein family (BET). BET proteins interact with chromatin modifiers and act as super-enhancers by binding to acetylated lysine residues on histones, recruiting transcriptional activator complex P-TEFb and facilitating activation of RNA polymerase II, resulting in transcription of target genes.^310,311^ BRD4 is a member of the BET family that is frequently upregulated in various cancers and has recently attracted therapeutic interest for its demonstrated regulatory roles in glioblastoma.^312–316^ BRD4 is known to regulate expression and stabilise protein levels of the proto-oncogene MYC.^311,314^ Furthermore, BRD4 activity is believed to be closely related to PI3K/AKT signalling,^317^ which is unsurprising considering the significance of this pathway in promoting MYC activity.

Histone methyltransferases, such as the catalytic enzyme EZH2 (enhancer of zeste 2) which acts within the polycomb repressive complex 2 (PRC2), alter gene expression by methylating histones, typically resulting in repressed gene expression.^318^ A meta-analysis of 575 glioma patients reported that EZH2 overexpression was associated with poor OS and PFS.^319^ EZH2-mediated methylation is known to suppress expression of *PTEN*,^320^ as well as directly methylate and activate NFκβ in glioblastoma.^321^ Regulation of EZH2 can occur by AKT-mediated phosphorylation,^322^ demonstrating an intricate link between EZH2 activity and PI3K signalling. EZH2 acts as a subunit of PRC2 and mediates H3K27 methylation. This mechanism is especially relevant to H3K27-altered DMG, a rare but lethal paediatric HGG. This particular alteration inhibits PRC2 activity and results in a global reduction in H3K27 methylation in a mouse model of DMG tumourigenesis and in human DMG cells, with the exception of retained H3K27me3 on the tumour suppressor *CDKN2A* locus, potentially due to EZH2 activity.^323^ Thus, EZH2 has been suggested as a potential therapeutic target in DMG.

Histone acetylation and the balance between HDACs and histone acetyltransferases play a vital role in chromatin accessibility and intrinsic plasticity. Deacetylation of histone proteins promotes a closed chromatin conformation and, therefore, reduced expression of tumour suppressors. With three classes (class I, class IIa and class IIb) and nine HDAC proteins, the complexity of their involvement in glioblastoma pathogenesis remains to be fully elucidated. Somatic mutations in *HDAC* genes are associated with specific DNA methylation subtypes in glioblastoma, demonstrating a potential link between DNA methylation and histone acetylation status.^32^

More specifically, pre-clinical models of glioblastoma have supported the role of HDAC6 in promoting glioma tumour cell proliferation.^324–326^ HDAC6 deacetylates α-tubulin to support microtubule-dependent cell motility^327^ and also plays a role in EGFR turnover.^328–330^ Direct phosphorylation of HDAC6 on site Tyr570 by EGFR may suppress HDAC6 activity and control a negative feedback loop associated with EGFR endocytosis and degradation.^329^ In support of this, HDAC6 over-expression has been shown to stabilise EGFR in glioblastoma cells in vitro.^324^ Moreover, ERK-mediated phosphorylation on serine 1035 promotes HDAC6 activity and, through the EGFR-MAPK signalling axis, promotes cell migration.^331^ Increased HDAC6 activity may also be a mechanism of therapeutic resistance in tandem with EGFR activation.^324^ There is pre-clinical evidence to support the concept of combining HDAC inhibitors with EGFR inhibitors as a potential therapeutic strategy.^332^ Whether this combination approach would result in clinical benefit to glioblastoma patients has yet to be explored, but may be considered in future treatment strategies, particularly in patients with EGFR amplification. Finally, AKT-mediated inhibition of GSK3β may also promote HDAC6 deacetylase activity, demonstrating the interconnected role of RTK signalling in HDAC6 regulation.

## Targeting epigenetic mechanisms

### Small molecule DNMT inhibitors

Two small molecule inhibitors of DNMTs, 5-azacytidine and decitabine, have demonstrated anti-tumour effects in pre-clinical glioblastoma models.^333–337^ 5-azacytidine has been explored in clinical trials in larger cohorts of patients with solid tumours and other malignancies (NCT02223052 and NCT03684811). However, more recently, results from a phase I/II clinical trial reported that none of the *IDH-*mutant glioma patients treated with azacytidine in combination with mutant IDH1 inhibitor, olutasidenib, had a clinical response to treatment and all patients progressed within 10 weeks (Table 2; NCT03684811). Decitabine has only been considered in combination with a dendritic cell vaccine in paediatric HGG and other CNS tumours (NCT02332889). This treatment strategy has demonstrated some success in paediatric neuroblastoma patients.^338,339^ Decitabine increases neoantigen expression in glioblastoma cells in vitro and promotes T cell activation and neoantigen-specific killing of tumour cells in a patient-specific manner.^337^ Thus, decitabine treatment may sensitise glioblastoma patients to immunotherapy, however, this has yet to be explored in the clinical setting.

**Table 2:** Epigenetic targeted therapy in GBM clinical trials

Therapy	Target	Phase	Indication	No. Patients	Treatment	mOS (weeks)	mPFS (weeks)	PFS6 (%)	PFS12 (%)	Best response to treatment				Ref
										PD	SD	PR	CR	
Azacytidine	DNMT1	I/II	IDH-mutant glioma	5	Azacytidine + Olutasidenib		8.29					0		NCT03684811
Trotabresib	BET	I	Newly diagnosed	18	Trotabresib + RT/TMZ		33.1	57.8		2	14		1	^344^
Belinostat	Pan-HDAC	II	Newly diagnosed	13	Belinostat + RT/TMZ	80.5	40.5	84						^348^
Panobinostat	Pan-HDAC	II	Recurrent	24	Panobinostat + Bevacizumab	39.2	21.8	30.4		3	14	7	0	^349^
Vorinostat	Pan-HDAC	I/II	Newly diagnosed	107	Vorinostat + RT/TMZ	70	34.8							^350^
		II	Recurrent	66	Vorinostat	24.8	48.7	15.2				2		^351^
		II	Recurrent	40	Vorinostat + Bevacizumab	45.2	16.1	30				9		^352^
		II	Recurrent	44	Vorinostat + Bevacizumab	33.9	16.1	26	10					^354^
		I/II	Recurrent	39	Vorinostat + Bevacizumab + TMZ	54.4	29.1	53.8	20.5		19	15	2	^353^
		II	Recurrent	37	Vorinostat + Bortezomib	13.9	6.5	0		32		1		^355^
Valporic acid	Class I/IIa HDAC	II	Newly diagnosed	37	Valporic acid + RT/TMZ	129	45.7	70	38					^362^

*CR* complete response, *BET* bromodomain and extraterminal-containing protein family, *DNMT* DNA methyl transferase, *HDAC* histone deacetylase, *mOS* median overall survival, *mPFS* median progression-free survival, *PD* progressive disease, *PFS6* progression-free survival at 6 months, *PFS12* progression-free survival at 12 months, *PR* partial response, *RT* radiotherapy, *SD* stable disease, *TMZ* temozolomide

### Small molecule BET inhibitors

Extensive pre-clinical studies have shown that BET inhibitors, including JQ1,^317,340^ OTX015 (MK-8628 or birabresib),^341^ and I-BET151,^312,313^ reduce glioblastoma cell proliferation, inhibit cell cycle progression, and reduce tumour growth in vivo. Furthermore, treatment with OTX015 has shown synergistic effects in combination with TMZ in orthotopic glioblastoma tumours in vivo.^341^ However, a phase II clinical trial of OTX015 as a monotherapy in recurrent glioblastoma patients was terminated due to lack of clinical activity (NCT02296476). Another BET inhibitor, trotabresib, has resulted in some clinical response in other solid tumours.^342^ Trotabresib is the only BET inhibitor to sufficiently cross the BBB and penetrate brain tumour tissue in clinical HGG patients to date.^343^ The combination of trotabresib plus RT/TMZ has been investigated in newly diagnosed glioblastoma patients with some promising preliminary results, including two patients who demonstrated either a partial or complete response for over 50 weeks (Table 2).^344^ Further validation of these results is pending as this clinical trial continues to recruit glioblastoma patients (NCT04324840).

### Small molecule EZH2 inhibitors

The only clinically relevant EZH2 inhibitor is tazemetostat (EPZ6438), which has been approved for advanced epithelioid sarcoma patients^345^ and is currently under investigation in several clinical trials for lymphoma (NCT05228158, NCT05467943, NCT04224493, NCT05713110, NCT05627245), as well as other solid tumours and haematological malignancies (NCT05023655, NCT05627232, NCT04241835). Tazemetostat has not yet been investigated clinically for glioblastoma or DMG, likely due to the inconclusive results from pre-clinical models. In one study of a pre-clinical model of H3 K27-altered DMG, H3K27M-expressing cells and tumours were responsive to EZH2 inhibition by tazemetostat treatment in vitro and in vivo.^323^ In contrast, another more recent study has shown that EZH2 plays a tumour suppressor role in DMG and that EZH2 inhibition does not reduce DMG tumour burden in mice in vivo.^346^ However, other studies report conflicting results showing that tazemetostat does not inhibit paediatric HGG/DMG regardless of H3 mutation status^346,347^ and that EZH2 may even act as a tumour suppressor in DMG.^346^ Furthermore, paediatric HGG H3-wildtype cells positive for H3K27 methylation, which may frequently carry *TP53* mutations, did not respond to tazemetostat in vitro.^323^ Similarly, adult glioma neural stem cells that harbour *TP53* mutations are often associated with *CDKN2A* expression,^31^ and like paediatric HGG cells, did not respond to tazemetostat in vitro.^323^ Until the discrepancies surrounding which subtype of HGG patients would benefit from EZH2 inhibition are resolved, the use of tazemetostat in clinical trials for glioblastoma and other HGGs, warrants caution.

### Small molecule HDAC inhibitors

Since HDACs have significant connections with pro-tumoural cell signalling pathways, such as EGFR and PI3K, HDAC inhibitors have been given clinical attention for their ability to reset the epigenetic profiles that regulate cancer hallmarks. Pan-HDAC inhibitors include balinostat, panobinostat, and vorinostat. Balinostat has been tested in newly diagnosed glioblastoma patients in combination with RT/TMZ^348^ and panobinostat has been trialled in combination with bevacizumab in recurrent glioblastoma patients (Table 2).^349^ However, neither of these studies reported improvements in clinical outcomes, compared with their respective historical controls. Vorinostat has been more exhaustively examined in clinical trials for newly diagnosed glioblastoma patients in combination with RT/TMZ,^350^ as a monotherapy for recurrent glioblastoma^351^ or in combination with bevacizumab,^352–354^ or bortezomib^355^ for recurrent glioblastoma patients none of which showed clinical benefit. Only one study of vorinostat in combination with bevacizumab and TMZ met their primary end-point, with a PFS rate greater than 50% in recurrent glioblastoma patients (Table 2). However, this rate was not statistically significant to that of a previous report of bevacizumab-treated patients.^353,356^

Anti-epileptic drugs have HDAC-inhibiting activity and have been shown to sensitise brain cancer lines to RT in pre-clinical models.^357^ Of particular interest is valproic acid, a selective inhibitor for class I/IIa HDACs.^358,359^ There is substantial research to support the multi-faceted roles of valproic acid in inhibiting cancer hallmarks and its synergistic effect with other therapeutic agents in pre-clinical glioblastoma models.^360,361^ Newly diagnosed glioblastoma patients treated with valproic acid in combination with RT/TMZ had a median OS time of 129 weeks (Table 2).^362^ While this appears to be a substantial improvement in the survival of glioblastoma patients, these results are from a small study population and were not statistically compared to historical controls, so further research into this potentially beneficial combination is warranted. It is likely that the HDAC-inhibiting action of valproic acid increases the availability of target DNA to alkylating TMZ, enhancing its efficacy. In support of this, two meta-analyses including more than 2000 glioblastoma patients demonstrated that valproic acid treatment prolonged survival, compared with patients managed by standard care.^363,364^

Unfortunately, in children with HGG tumours, including DMG and glioblastoma, the addition of valproic acid to RT and maintenance bevacizumab showed little clinical benefit.^365^ However, four of the paediatric glioblastoma patients with constitutional mismatch-repair deficiency (MMRD) had prolonged OS and PFS, relative to patients with other types of HGGs, including a single patient who sustained a complete response for 24 months.^365^ These data suggest that valproic acid may provide unique benefit to this subset of glioblastoma patients. Larger clinical trials are required before valproic acid may be considered as an addition to standard care regimes. A phase III international clinical trial is currently recruiting to assess the clinical benefit of valproic acid with RT/TMZ for HGG in children (NCT03243461).

Isoform-specific HDAC inhibitors have not yet entered clinical trials for glioblastoma, however, several small molecule inhibitors for HDAC6 have been developed and studied mostly in the context of neurodegenerative diseases.^366–370^ HDAC isoform-specific inhibitors may be less toxic with fewer off target effects and fewer severe adverse effects than pan-HDAC inhibitors. Small molecule HDAC6 inhibitors including KA2507 (NCT03008018) and JBI-802 (NCT05268666) are in early phase clinical trials for advanced solid tumours.

## Obstacles in using targeted therapies in glioblastoma

The therapeutic strategies above have essentially failed. Indeed, over 1000 brain cancer clinical trials have been undertaken;^371^ but these trials have not significantly improved patient outcomes.^372^ Exhaustive genetic profiling using bulk sequencing,^32^ single cell sequencing,^373^ and epigenetic analysis^374^ have identified potential vulnerabilities, but once again, none have spawned successful new treatments. The reasons for these failures are multi-factorial, but often drugs selected for glioblastoma trials have been repurposed from use in extra-cranial malignancies, with only superficial testing in glioblastoma models that fail to address key disease-specific obstacles.^372^

**Fig. 2:**
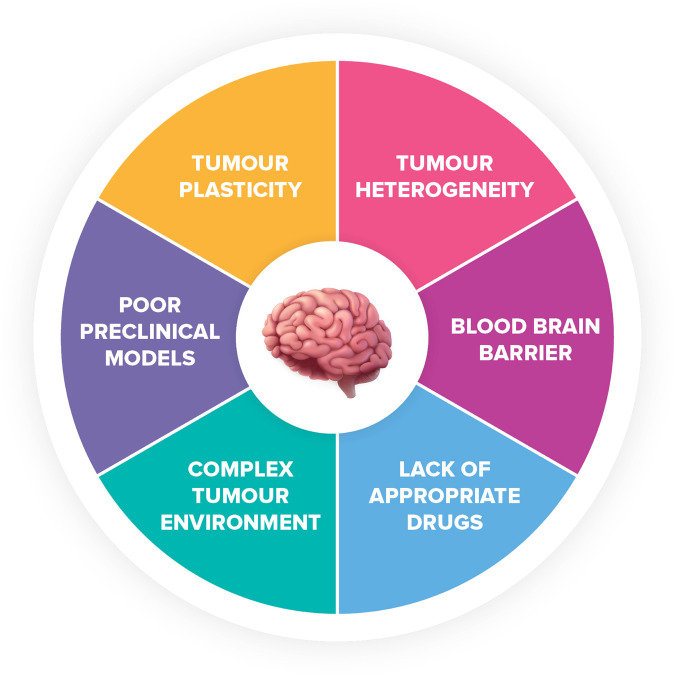
Key obstacles in developing new treatments for glioblastoma

The glioblastoma-specific obstacles that have prevented the translation of successful therapeutics from other cancers into improved glioblastoma outcomes are multi-factorial (Fig. 2). Primary among these obstacles is the BBB, which is a major hinderance to the delivery of drugs to the glioblastoma site.^375^ The dynamic tumour microenvironment composed of neurons, microglia, astrocytes, and immune cells likely contributes to therapeutic resistance. Furthermore, glioblastoma cells are derived from neural cells, and therefore, show a higher level of plasticity compared to most tumour types; a trait that allows them to adapt rapidly to therapeutic treatment.

The combination of obstacles highlighted in Fig. 2 illustrates the complications in developing new drugs for the treatment of glioblastoma. Potential strategies for addressing these obstacles have been clearly articulated in a recent position paper “Challenges to curing primary brain tumours” written by the world-leaders in brain cancer.^16^ Some obvious, but nonetheless challenging, strategies include acquiring drugs that cross the BBB or developing methods that open the BBB. There are considerable efforts in this area.^375^ Identifying drugs that disrupt the glioblastoma cell/neuron interaction might provide new treatments or enhance existing drugs. Strategies on how we could achieve this are being developed.^376^ Tackling the problem of glioblastoma cell plasticity is a particularly tough challenge, that almost certainly requires novel combination of drugs^371^ including those that might prevent glioblastoma cell plasticity.

More broadly there is strong agreement that innovative and quicker trials in glioblastoma are required.^16,372^ Adaptive clinical trials, combined with robust biomarkers, is especially appealing in the glioblastoma space. These trials can rapidly identify ineffective treatments and expand therapies that show initial promise.^16,372^ Equally important, such clinical trials can remain open indefinitely rather than opening and closing.^372^

Maybe our current strategies, like targeting kinases, will never lead to effective treatments. In the very least, their use in the clinic is likely to only be beneficial when combined with other anti-cancer agents as part of a focused precision medicine approach. Therefore, below we discuss other possible strategies based on metabolism, immunotherapy, and targeting the cell of origin as other avenues that may be employed in future therapeutic strategies.

## Targeting metabolism

While cancer metabolism has become a major focus in recent years, progress in therapeutically targeting metabolism has so far been disappointing. Many chemotherapeutics have metabolic effects, however clinical trials of agents such as the folate antagonist methotrexate have not been successful in glioblastoma.^377^ Most of the glioblastoma signalling pathways discussed in this review are directly or indirectly linked with metabolic pathways. Nutrient uptake and metabolism facilitate the increased synthesis of proteins, nucleotides, and fatty acids necessary for cell division. Despite the tantalising therapeutic potential of targeting tumour metabolism, these discoveries have proven difficult to translate into improved patient outcomes.

Glioblastoma cells have a high metabolic need, with an inherent requirement for glucose, as they are unable to use ketone bodies for growth.^378^ These discoveries have led to a number of clinical trials of dietary interventions, including ketogenic diets, in an attempt to starve the cancer cells (NCT00575146, NCT04730869, NCT01535911, NCT03451799, NCT01754350, NCT02046187, NCT03160599). ERGO (NCT00575146) was a small pilot study which established the feasibility and safety of a ketogenic diet in glioblastoma,^379^ consistent with other small prospective and retrospective studies.^380–382^ ERGO2 (NCT01754350) examined a ketogenic diet and intermittent fasting, however no significant difference in PFS was observed when compared to control diet in patients receiving re-irradiation for recurrent brain tumours.^383^ A number of these dietary interventions are also being tested in combination with the biguanide metformin in an attempt to enhance the inhibitory effects of ketones by targeting glucose utilisation. Metformin is an oral anti-diabetic drug that is well-tolerated and commonly used for the treatment of type 2 diabetes. Many pre-clinical studies in a range of cancers, as well as retrospective clinical trials, have shown the potential anti-cancer effect of metformin.^384^ This led to a number of clinical trials investigating the efficacy of metformin in glioblastoma patients (NCT03243851, NCT03151772, NCT02780024, NCT01430351, NCT04945148). However, a recent analysis of 1731 glioma patients from three separate clinical trials in glioblastoma patients showed no significant increase in either PFS or OS; in fact a trend toward decreased glioblastoma survival at baseline for metformin monotherapy was observed.^385^ Additional data are required to determine whether there are biomarkers that may select for metformin efficacy or combination treatments with other agents (or diets) that may provide significant benefit to patients. For example, a phase I clinical trial (NCT02149459) of 13 patients determined that a modified Atkins diet and 850 mg metformin twice daily was well-tolerated.^386^ Phase II clinical trials are currently recruiting (NCT04691960), including a combination therapy with the PI3K inhibitor paxalisib (NCT05183204). Maximum tolerated dose and dose-limiting toxicities have been determined for metformin in combination with memantine, an inhibitor of N-methyl-D-aspartate (NMDA)-type glutamate receptors, as well as mefloquine, an autophagy inhibitor.^387^

Autophagy, which is induced by cellular stress, causes proteins and other cellular content to be degraded in lysosomes, which facilitates recycling of cellular contents for protein synthesis and other metabolic pathways.^388^ Autophagy is a critical metabolic pathway that can provide the necessary metabolites to ensure tumour cell survival during periods of stress. Another autophagy inhibitor, chloroquine, has been tested in both pre-clinical glioblastoma studies as well as dose evaluation in a clinical trial.^389^ Pre-clinical studies have shown that chloroquine efficacy may be enhanced in EGFR-mutant glioblastoma, due to increased dependency on autophagy. These data suggest that autophagy inhibitors may act synergistically with EGFR-targeting agents.^390^

A metabolic shift to glucose oxidation in glioblastoma results in increased production of reactive oxygen species (ROS), including mitochondrial superoxide, which requires glioblastoma cells to upregulate redox pathways such as glutathione synthesis.^391^ Redox balance can be targeted by either increasing ROS-induced cellular damage, or blocking the intrinsic regulatory redox pathways from regulating ROS-induced damage. For example, BPM31510 is a drug-lipid conjugate nanodispersion of ubidecarenone (oxidised CoQ10) that specifically increases mitochondrial superoxide in glioblastoma tumour cells,^392^ which is currently being tested in a clinical trial for newly diagnosed glioblastoma patients (NCT04752813). Another mechanism used to target redox metabolic pathways is inhibition of the xCT/SLC7A11 cystine/glutamate exchanger, which is increased in glioblastoma.^391^ Blocking cystine uptake (and glutamate export) using the anti-inflammatory drug sulfasalazine decreases the levels of cysteine required for glutathione production and inhibits glioblastoma cell growth.^391^ However, a European clinical trial (ISRCTN45828668) of sulfasalazine in eight glioblastoma patients was terminated after interim analysis due to a lack of efficacy and a high incidence of adverse events.^393^ Despite this, a current clinical trial is examining sulfasalazine as a means of targeting glutamate metabolism, rather than redox balance, in glioblastoma (NCT05664464). This trial is using a three-pronged approach to blocking glutamate, combining sulfasalazine inhibition of glutamate export, gabapentin inhibition of glutamate production (via branched-chain aminotransferase activity) and memantine to inhibit glutamate receptor activation.

Dichloroacetate (DCA) is another repurposed metabolic agent currently being tested in glioblastoma, due to its ability to switch pyruvate usage from aerobic glycolysis to glucose oxidation in the Krebs cycle through inhibition of PDK activity.^394,395^ PDK is a metabolic enzyme that is increased to promote the Warburg effect, which normally inhibits the activity of mitochondrial pyruvate dehydrogenase complex (PDC), thereby blocking the conversion of pyruvate to acetyl-CoA and subsequent entry into the Krebs cycle and promoting pyruvate conversion to lactate for continued NAD^+^ synthesis and glycolytic flux.^395^ A small first-in-human trial of five glioblastoma patients showed that DCA was safe and well-tolerated, with some putative therapeutic benefit in some patients.^396^ A recommended phase II dose (6.25 mg/kg BID) was determined in 23 patients with advanced solid tumours including breast (*n* = 3), colorectal (*n* = 6), head and neck (*n* = 3) and lung (*n* = 5),^397^ with a new clinical trial currently underway in glioblastoma (NCT05120284). DCA also has the potential to work in combination with EGFR inhibition by blocking EGFR stimulation of acetyl-CoA via an alternative PDC pathway in the nucleus. EGFR activation has been shown to stimulate a shift from mitochondrial activity to increased nuclear expression of PDC and de novo production of nuclear acetyl-CoA, which is required for histone acetylation levels and cell cycle progression, suggesting a role for EGFR in regulating metabolic activity via an alternative PDC pathway.^398^ Furthermore, some studies have demonstrated a correlation and functional link between PDK expression and EGFR expression in glioblastoma.^399,400^ Therefore, a combination of DCA and EGFR inhibitors would simultaneously block PDK to reverse the Warburg effect and reduce the production of high NAD^+^ levels needed for cell growth, as well as decrease the nuclear translocation of PKC and cell cycle progression that would consequently be promoted through DCA treatment. This combination approach has shown some synergistic effects in non-small-cell lung cancer cell lines,^401^ however, this has yet to be tested in glioblastoma.

## Immunological targets

In recent years, monoclonal antibodies targeting immune checkpoints have been approved by the FDA for the treatment of various cancers including colorectal, gastric, and various haematological malignancies.^402^ These antibodies promote T cell activation by interfering with inhibitory signalling caused by receptor-ligand interactions between effector T cells and tumour cells, such as PD1-PDL1, CD80-CTLA4 and CD86-CTLA4. Upon activation, CD4^+^ T cells secrete cytokines that modulate, enhance, and fine-tune local anti-tumour immune responses. CD8^+^ T cells directly lyse tumour cells when their T cell receptor recognises abnormal peptides presented on tumour cell major histocompatibility complex (MHC) class I receptors. The importance of T cells in killing cancer has been long recognised, with tumour-infiltrating lymphocytes (TILs) correlating with greater survival across various cancer subtypes, including ovarian,^403^ breast,^404^ and colorectal.^405^

Although the most effective immunotherapies identified against cancer to date utilise the actions of T cells, brain cancers are inherently immunosuppressive and, as mentioned previously, have a low TMB.^299^ The latter point is especially significant given the FDA approval of the PD-1 immune-checkpoint inhibitor, pembrolizumab, for patients with any cancer that has high TMB, as this would exclude many glioblastomas. The exception to this is in children with biallelic MMRD, which often results in glioblastoma characterised by high TMB and where durable responses to immune checkpoint inhibition have been reported.^406^ Counter-intuitively, multiple recent studies have shown that there is a trend toward longer survival for patients with glioblastoma who had lower TMB following treatment with immune checkpoint blockade.^407–409^

Neoadjuvant pembrolizumab therapy may restore TIL function in adults with recurrent glioblastoma, resulting in a survival benefit compared with adjuvant administration in a small population of patients (Table 3).^410^ However, nivolumab, another anti-PD-1 therapy, has failed to yield clinical benefit in large randomised patient cohorts of both newly diagnosed and recurrent glioblastoma (Table 3).^411–413^ To investigate the potential mechanisms underlying this treatment failure, Lee *et al*. administered neoadjuvant pembrolizumab to patients with recurrent glioblastoma prior to surgical debulking and examined the intratumoural immune microenvironment.^414^ They showed that immune-checkpoint inhibition altered professional antigen presenting cells and increased T cell infiltration; however, these T cells exhibited features of exhaustion. Furthermore, tumour-associated microglia and macrophages remained the most abundant immune cells in adult glioblastoma and may contribute to an immune suppressive environment, leading to therapy resistance.

**Table 3:** Immune targeted therapy in GBM clinical trials

Therapy	Target	Phase	Indication	No. Patients	Treatment	mOS (weeks)	mPFS (weeks)	PFS6 (%)	Best response to treatment				Ref
									PD	SD	PR	CR	
Pembrolizumab	Anti-PD-1	Pilot	Recurrent	16 neoadjuvant; 16 adjuvant	Pembrolizumab + surgery	59.6; 32.6	14.2; 10.4						^410^
Nivolumab	Anti-PD-1	III	Newly diagnosed (methylated MGMT)	358	Nivolumab + RT/TMZ	125.7	46.1						^411^
		III	Newly diagnosed (unmethylated MGMT)	280	Nivolumab + RT	58.3	26.1	50.5					^413^
		III	Recurrent	347	Nivolumab	42.6	6.5		107	33	10	2	^412^
CAR T cell	GD2	I	Recurrent or Progressive	8	GD2-CAR T cells	40			3	1	4	0	^436^
	EphA2	Pilot	Recurrent	3	EphA2-CAR T cells	23		0	2	1	0		^430^
	EGFRvIII	I	Recurrent	10	EGFRvIII-CAR T cells	35.9				9	0		^432^
	EGFRvIII	Pilot	Recurrent	18	EGFRvIII-CAR T cells	30	5.7		16		0		^431^
	HER2	I	Recurrent	17	HER2-CAR T cells	48.3	15.2	35.3	9	7	1	0	^433^
	IL13Ra2	Pilot	Recurrent	3	IL13(E13Y)-zetakine CAR T cells	47.9							^435^
	IL13Ra2	I	Recurrent	1	IL13BBζ–CAR T cells		32.6					1	^434^

*CAR* chimeric antigen receptor, *CR* complete response, *EphA2* ephrin type-A receptor 2, *EGFR* epidermal growth factor receptor, *HER2* human epidermal growth factor receptor 2, *IL13Rα2* interleukin 13 receptorα2, *mOS* median overall survival, *mPFS* median progression-free survival, *PD* progressive disease, *PFS6* progression free survival at 6 months, *PR* partial response, SD stable disease

### Looking forward: new immune checkpoints for glioblastoma

Myeloid cells are reported to comprise up to 50% of some brain tumours.^415^ These glioma-associated macrophages are derived from brain-resident microglia and from hematopoietic stem cell-derived cells that infiltrate glioblastoma from the periphery. Microglia originate from primitive yolk sac progenitor cells that migrate to the developing brain and spinal cord early in foetal development. Once they reach the CNS, microglia differentiate and proliferate to become the resident macrophages of the brain.^416^ Notably, although brain-resident and peripheral myeloid cells share many features, including phagocytic activity and expression of markers such as CD45 and CD11b, they can play distinct roles in auto-immune-associated CNS diseases.^417^ In the context of glioblastoma, both tumour-associated microglia and tumour-infiltrating macrophages are capable of glioma cell phagocytosis.^418^ This, along with their abundance in gliomas has led many to investigate the potential of therapeutics that can re-direct these myeloid cells to act as anti-tumour effector cells.^418–420^

Macrophages typically recognise cells that should be targeted for degradation via exposure of “eat me” signals on the plasma membrane.^421^ The best-characterised of these is phosphatidylserine, which is normally on the inner plasma membrane but is flipped to the outer membrane of dying, dead, infected or injured cells, marking them for phagocytosis.^422^ Other “eat me” signals include calreticulin and sugars, such as galactose. Importantly, in normal homoeostasis these “eat me” signals are balanced by inhibitory “don’t eat me” signals to avoid uncontrolled phagocytosis, particularly of haematopoietic cells.

CD47, a crucial inhibitory protein, is expressed on the surface of most normal cells and interacts with SIRPα on macrophages.^423^ This interaction allows healthy cells to evade immune surveillance and avoid destruction.^424^ However, in cancer, CD47 is often overexpressed, enabling cancer cells to evade the immune system and proliferate unchecked. This immune evasion strategy is particularly effective for brain tumours because of the abundance of innate immune cells in the brain. Notably, almost all brain tumour types exhibit significantly elevated CD47 expression.^420^ As a result, CD47-targeted therapies have emerged to block the CD47-SIRPα interaction, enhancing immune recognition and enabling the immune system to attack cancer cells more effectively.

Several approaches are currently being investigated to target CD47, although none have been approved for clinical use to date. The majority of CD47-targeted therapies in development are monoclonal antibodies, but small molecule inhibitors have been gaining attention. Hu5F9-G4 is the most extensively studied monoclonal antibody targeting CD47. Also known as magrolimab, Hu5F9-G4 has shown promising results in pre-clinical studies as a potential brain cancer therapy. A landmark study by Gholamin, Mitra and colleagues performed a comprehensive in vivo analysis of the effect of Hu5F9-G4 against different paediatric brain cancers, including glioblastoma.^420^ These data were validated in vivo using the SU-GBM044 patient-derived cell line model of adult glioblastoma,^425^ proving that inhibiting the CD47-SIRPα interaction can improve the survival of mice with glioblastoma. Furthermore, pre-clinical data suggests that the actions of anti-CD47 antibodies can be further boosted using current standard treatments for glioblastoma. Gholamin, Mitra et al. demonstrated that when anti-CD47 (Hu5F9-G4) was combined with either irradiation or TMZ, phagocytosis of glioblastoma cells by peripheral blood mononuclear cell-derived macrophages was enhanced in vitro.^420^ Importantly, in vivo, the combination of RT with Hu5F9-G4 significantly improved survival in a patient-derived xenograft model of glioblastoma.^426^

Although these pre-clinical studies targeting CD47 in glioblastoma have shown promise, there have been no clinical trials to date. A first-in-human clinical trial for Hu5F9-G4 reported that the antibody was well-tolerated, however, patients with primary brain cancer or brain metastases were excluded.^427^ Ongoing trials are evaluating Hu5F9-G4 in combination with various agents, including with rituximab in B cell lymphoma, and although the numbers are small, this approach showed promising activity with no clinically significant safety events.^428^ Other trials are investigating Hu5F9-G4 in combination with cetuximab in colorectal cancers, in combination with azacytidine in treatment-naïve acute myeloid leukaemia (AML) and myelodysplastic syndrome, and in combination with the anti-PDL1 agent avelumab in patients with ovarian cancer (NCT02953782, NCT03248479, NCT03558139). Multiple other antibodies targeting CD47 or SIRPα are being investigated in clinical trial (recently reviewed^429^), but no clinical trials are investigating anti-CD47 in combination with RT in glioblastoma.

Despite this ongoing clinical activity, there are several potential challenges that are yet to be addressed. Adverse effects such as anaemia are probable and potentially exacerbated by the long half-life of antibody therapeutics, as CD47 expression is a known mechanism used by red blood cells to avoid macrophage-mediated phagocytosis. To address this challenge, small molecule inhibitors or therapeutic peptides capable of blocking the interaction between CD47 and SIRPα but with differing pharmacokinetic properties to antibodies are being developed,^429^ however, this research is early-stage and no drug candidates have reached clinical trials. Moreover, whether such agents can successfully cross the BBB has not been reported.

### Looking forward: cellular therapy

Cellular therapies, particularly chimeric antigen receptor (CAR) T cell therapy, hold great promise as immunotherapeutic approaches for brain tumours. These therapies utilise synthetic receptors to activate T cells independently of major histocompatibility complex (MHC), overcoming a common immune evasion mechanism employed by tumour cells. CARs are modular immune sensor receptors typically composed of an extracellular single chain variable fragment (scFv) derived from an antibody, which provides antigen specificity. They also possess a transmembrane domain for cell surface expression and intracellular costimulatory domains that recruit signalling proteins necessary for T cell activation. In CAR T cell therapy, the patient’s own T cells are acquired via apheresis, re-engineered ex vivo to acquire the ability to selectively kill tumour cells in an antigen-dependent manner and then transferred back into the patient. This approach differs from checkpoint blockade and tumour vaccine strategies that stimulate the patient’s endogenous T cell response. Indeed, the success of this strategy requires the identification of relatively cancer-specific targets on the cell surface.

Clinical trials investigating CAR T cell therapy for glioblastoma are targeting five antigens: EphA2,^430^ EGFRvIII,^431,432^ HER2,^433^ interleukin 13 receptorα2 (IL13Rα2),^434,435^ and GD2^436,437^ (Table 3). Early-phase results have been published for these targets, revealing some notable outcomes. While OS did not improve significantly across patient cohorts treated with CAR T cell therapy, a single patient who did not receive post-CAR glioblastoma treatment remained alive 59 months after EGFRvIII-CAR T cell therapy.^431^ Furthermore, some patients treated with HER2-CAR T cell therapy maintained a stable condition for up to 29 months.^433^ A patient receiving anti-IL13Rα2 CAR T cells demonstrated a complete response (determined by clinical and radiographic observations) for 7.5 months,^434^ suggesting promising potential for this immunotherapy in glioblastoma treatment. It is important to note that this patient was enrolled in an ongoing phase I clinical trial (NCT02208362), and received 16 intracranial injections of CAR T cells, highlighting the need for multiple doses to achieve anti-tumour efficacy in the brain, unlike CAR T cell trials targeting haematological cancers. Similarly, Majzner *et al*. recently reported transient clinical improvements in a paediatric study of four patients with DMG who received rounds of GD2 specific CAR T cell infusion, a therapy targeting a glycosphingolipid that is often highly expressed in brain cancer.^437^ A separate GD2 CAR T cell clinical study observed median overall survival of 10 months post infusion across a cohort of 4 children and 4 adults with glioblastoma.^436^ Here, pre and post infusion MRI comparison indicating reduced lesion sizes in 4 patients (2 adults and 2 children) supported partial responses following a single intravenous dose of autologous GD2 CAR T cells,^436^ highlighting that there is much more to learn about the cadence and route of CAR T cell administration. Various actively recruiting clinical studies investigating dosage and toxicities for treating patients with malignant gliomas using GD2 CAR T cell therapy (NCT04196413, NCT03423992 and NCT04099797) are yet to report findings. These clinical studies confirm that cellular therapies can penetrate the BBB,^432^ providing an advantage over small molecule drugs with limited capacity to access brain tumour cells. An emerging CAR T cell therapy target is B7-H3 (B7 homologue 3 protein, also known as CD276), which is a novel co-stimulatory molecule that modulates immune responses.^438,439^ Several clinical trials are recruiting to evaluate the effectiveness of B7-H3-CAR T cell therapy in recurrent glioblastoma patients (NCT05241392, NCT05474378, NCT04077866, NCT04385173, NCT05366179). Further research will provide valuable insights into the administration protocols and routes necessary for optimal therapeutic outcomes.

CAR T cell therapy may be challenging in glioblastoma because this type of cancer is known to be highly heterogenous^440,441^ and can alter gene expression patterns in response to standard treatment.^442^ Moreover, in several trials, despite tumour regression being observed, the patients all ultimately relapsed, suggesting that CAR T cell efficacy can be limited by antigen escape in a clinical setting. To improve treatment efficacy, various clinical trials are investigating whether glioblastoma patients would benefit from a combination therapy, either as two immune checkpoint inhibitors or a CAR T cell infusion with neoadjuvant or adjuvant checkpoint blockade.^441^ Alternatively, multi-antigen targeting cellular immunotherapies are potential solutions for treating cancers with high intra-tumoural heterogeneity.^443,444^ A clinical trial is currently underway for a dual-antigen T cell therapy targeting both EGFR/EGFRvIII and IL13Ra2 in recurrent glioblastoma (NCT05168423). Brainchild-04 (NCT05768880) will be another, paediatric-focused phase I clinical trial that will be the first to use a quad-specificity multi-antigen-targeted CAR T cell therapy to treat DMG.

**Fig. 3:**
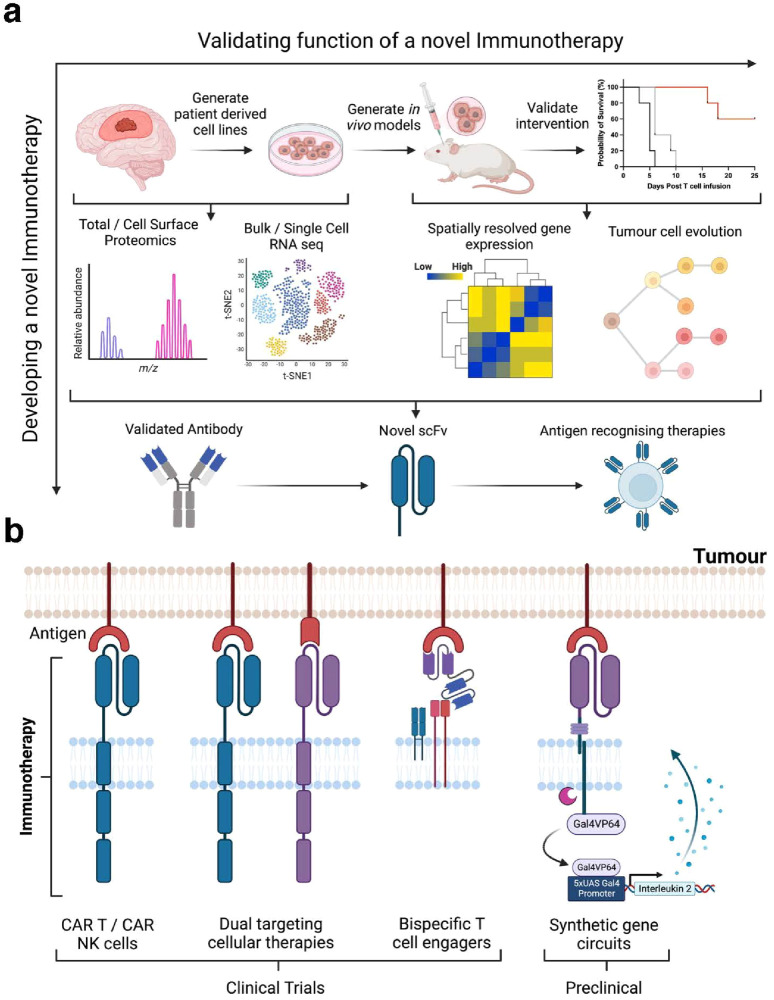
Overview of immunotherapy development and application for treating adult glioblastoma. **a** Primary tumour samples from patients are used to create in vitro and in vivo glioblastoma models to test novel interventions against. For example, survival probability of mice that are intracranially injected with cultured glioblastoma cells and treated with the novel therapy. From patient tumour samples and patient derived cell lines, multi-omics analyses enable interrogation of tumour profiles (e.g. gene transcription, protein expression, protein mis-localisation). Spatio-temporal analyses of tumour growth in vivo improves understanding of tumour evolution and cell or origin. Comprehensive investigation of the underlying biology informs antigen candidate selection and development of novel scFv’s from validated antibodies. **b** Novel scFv’s are engineered into various immunotherapies. Bispecific T cell engagers and cellular therapies including CAR T cells, CAR Natural Killer cells and dual targeting (multivalent) CAR T cells for glioblastoma are being evaluated in clinical trials. Anti-tumour responses towards glioblastoma such as cytokine secretion and CAR translation have successfully been induced using synthetic gene circuits in preclinical models. Figure created on BioRender.com. Chimeric antigen receptor, CAR; scFv, short chain variable fragments. Figure created with BioRender.com

Validation of surface antigens expressed on glioblastoma cells through pre-clinical studies is crucial for the advancement of novel CAR T cell therapies in clinical trials. Therefore, it is necessary to investigate the surfaceome of patient-derived tumour cells using various molecular techniques to select appropriate antigens for CAR T cell targeting. While characterising the transcriptome provides key insights into the aberrant signalling pathways that promote glioblastoma growth, it has limited utility for CAR T cell development. Transcriptome findings do not always correlate with protein expression, and gene expression alone lacks information about protein localisation. Proteins that are mis-localised to the plasma membrane of tumour cells are promising neoepitopes to target with CAR T cells, as shown pre-clinically with a CAR T cell targeting the mitochondrial protein GRP78 to treat AML.^445^ Targeting such neoepitopes reduces the risk of on-target off-tumour cytotoxicity. Furthermore, it is important to determine whether non-protein antigens are overexpressed in tumours. Relying solely on mass spectrometry to interrogate total and cell surface proteins may overlook potential immunotherapeutic targets. Therefore, antigen discovery pipelines that employ a multi-omics approach, including gene expression, total protein and cell surface protein expression, as well as lipidome analyses, have proven to be the most useful for designing novel CAR T cells (Fig. 3).^446–448^

The synthetic Notch (synNotch) platform can be expressed in T cells and uses recognition of a single antigen to drive transcription of a transgene, allowing for localised anti-tumour responses within the tumour microenvironment.^449^ This can include CAR expression, cytokine secretion,^450^ or production of bispecific T cell engagers (Fig. 3).^451^ The synNotch platform has been successfully applied to treat intracerebral glioblastoma PDX mouse models. Using myelin oligodendrocyte glycoprotein detection, it induced expression of an EGFRvIII targeting CAR in primary human T cells, resulting in greater tumour killing, reduced exhaustion, and improved safety over conventional CAR T cells.^452^

In the paediatric space, recent studies have shown promising results with GD2-targeted therapy for DMG. Three out of four children exhibited clinical benefit following intravenous administration followed by intracerebroventricular administration.^437^ Importantly, patients experienced better quality of life due to improved motor function after treatment,^437^ suggesting that effective therapy may partially reverse the debilitating symptoms caused by malignant gliomas. The route of CAR T cell delivery may affect efficacy in both paediatric and adult patients. Currently, only IL13Rα2-specific CAR T cells have been administered intracranially to treat adult glioblastoma.^434,435^ While there is much work still required to see long-term survival benefits for adults with glioblastoma, or more broadly malignant gliomas, research spanning tumour characterisation, pre-clinical validation and clinical trials highlights the strong potential that cellular therapy and combined immunotherapy should be implemented to treat this disease.

## Future perspective: the cell-of-origin to drive personalised medicine in glioblastoma

**Fig. 4:**
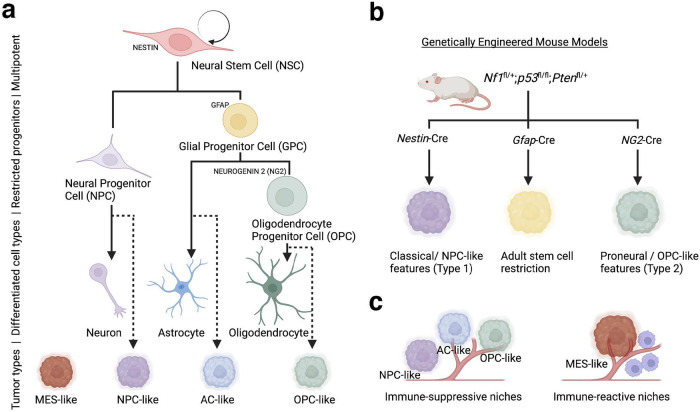
Cell-of-origin implications in glioblastoma tumour cell states. **a** Association of neural cell hierarchy with tumour cell states based on recent single cell transcriptomics analysis. The multipotent neural stem cell (NSC) gives rise to lineage-restricted neural progenitor cells (NPC) and glial progenitor cells (GPC). GPCs give rise to oligodendrocyte progenitor cells (OPC) and the restricted progenitors produce differentiated neurons, astrocytes and oligodendrocytes in the normal developing brain, however glioblastoma tumour cells display transcriptional properties closely paralleling these cell states. The mesenchymal-like (MES-like) tumour cell state has no direct parallel to the neural cell hierarchy however has similar properties to radial glia.^36^ **b** Genetically engineered mouse models carrying *Nf1*^fl/+^;*p53*^fl/fl^;*Pten*^fl/+^ alleles develop glioblastoma, with different tumour phenotypes depending on the originating cell-of-origin, directed by Cre-recombinase expression. **c** Tumour cell states are associated with characteristic immune infiltration, with MES-like displaying the highest degree of infiltration across multiple sequencing studies. Figure created with BioRender.com

Experimental model systems, such as genetically engineered mouse models, have been used to model glioblastoma development and provide evidence for the impact of cell-of-origin on glioblastoma subtype.^453–455^ A classic example is the combined loss-of-function mutations in *Nf1*, *p53* and *Pten* (*Nf1*^fl/+^;*p53*^fl/fl^;*Pten*^fl/+^) in glial cells.^456,457^ Glial cell specificity can be driven by the expression of Cre recombinase, which directs the deletion of floxed alleles. Promoters that are specific to glial cell expression, such as *Nestin* (neural stem and progenitor cells), *Gfap* (adult neural stem cells and astrocytes) and *Neurogenin2* (oligodendrocyte progenitor cells) have been engineered to drive CreERT2 recombinase expression, restricted in cells with an active promoter and specifically activated following treatment with tamoxifen. Glioblastoma tumours develop in *Nf1*^fl/+^;*p53*^fl/fl^;*Pten*^fl/+^ mice driven in neural progenitor cells by *Nestin*-Cre,^456^ adult stem cells by *Gfap*-Cre,^458^ and restricted oligodendrocyte progenitor cells by *NG2*-Cre (Fig. 4).^455^ This suggests that both stem and restricted progenitor cells in multiple glial cell lineages can give rise to glioblastoma development with this combination of genetic alterations.

However, when tumours are induced in different lineages at the same age, they give rise to glioblastoma of different tumour subtypes: Type 1 driven by *Nestin*-Cre with features of classical/NPC-like and Type 2 driven by *NG2*-Cre with features of proneural/OPC-like tumour states.^455,459^ Molecular features of these tumours are tightly linked to their cell-of-origin, characterised by the expression of *EGFR* and *ERBB2*, but also revealed cell-type specific therapeutic vulnerabilities, such as to the ERBB2 inhibitor dasatinib in Type 2 OPC-like tumours.^455^ These pre-clinical models define the imprinting that the cell-of-origin has on the tumour, thus providing insights into the origins of heterogeneity and plasticity.

Indeed, this cell-of-origin and subtype-driven heterogeneity and plasticity impacts the ability to target glioblastoma with personalised therapy.^460^ Neftel et al. have shown that the overexpression of driver oncogenes for each tumour subtype can shape the distribution of cell states. For example, EGFR overexpression induces AC-like programmes in neural progenitor cells.^35^ Thus, key oncogenic drivers are intricately linked to tumour cell state and can be inhibited and manipulated to target the tumour or drive it to alternate cell states. Such targets include EGFR inhibition in AC-like dominant tumours, CDK4 inhibition in NPC-like tumours and PDGFRA inhibition in OPC-like tumours.^35^ Given the plasticity and heterogeneity of glioblastoma, combination therapy approaches using small molecule inhibitors targeting multiple subtypes would likely be required to realise personalised therapy for glioblastoma. Tumour subtyping not only identifies molecular targets, but may also guide the use of immunotherapies. To facilitate the effective use of immunotherapies, researchers have started to study the effect of tumour cell states on the composition of the immune compartment. By performing scRNAseq separately on the tumour core and periphery, Darmanis *et al*. found that microglia had a distinct pro-inflammatory role in the peritumoural space in contrast to macrophages found at the tumour core.^461^ These results have been extended using spatial transcriptomics on a large cohort of glioblastoma patients identifying an immune-reactive niche containing tumour cells spanning the mesenchymal and astrocyte-like states.^440^ Reanalysis of these data updated this model to show that the mesenchymal patches were infiltrated by monocytes and macrophages (Fig. 4),^462^ a finding that was validated in an independent analysis.^463^ Interestingly, the presence of macrophage-enriched neighbourhoods in glioblastoma tumours is associated with prolonged survival, suggesting that targeting macrophages in the clinical setting may be beneficial.^464^

New technologies, like scRNAseq and spatial transcriptomics, in combination with observations from experimental model systems are transforming the concept of cell-of-origin in glioblastoma to a complex cell state model involving considerable plasticity and unique capacity for self-renewal. Therefore, the notion that tumour-initiating cells in glioblastoma can be eliminated for considerable clinical benefit should be replaced with an updated approach of identifying one or multiple tumour cell states that are inducible and targetable by therapies or drug combinations.^465^ This search will be informed by scRNAseq with its ability to tease apart the molecular pathways that drive differentiation and confer tumourigenic potential in different tumour cell states. The addition of spatial transcriptomics to this process will identify opportunities to harness the patient’s immune system to eliminate a specific tumour cell state. Unlike existing therapies, these novel treatments are likely to represent truly personalised approaches. However, their effective use in clinical practice will require the incorporation of these new technologies to deliver precision medicine and to monitor the success of such therapeutic interventions in glioblastoma.

## Concluding remarks

We cannot keep using the same clinical strategies, they have not worked in glioblastoma. The section above describing CAR T-cells strategies offers a novel approach that is giving hope to the brain cancer community. Further research on the cell-of-origin will also hopefully provide novel therapeutic strategies. More generally, developing new strategies that enhance BBB penetrance are sorely needed.^16,372^ In addition, understanding and targeting key biological processes such as tumour heterogeneity, neuronal glioblastoma interaction, and immune response is vital.^16,372^ Finally, innovative trial designs that speed up the clinical assessment are urgently required, particularly those that use combinational approaches.^372^ Our knowledge of glioblastoma biology has grown exponentially in the past decade and the time has come to translate this information into improved clinical outcomes.
